# Are Suggested Hiking Times Accurate? A Validation of Hiking Time Estimations for Preventive Measures in Mountains

**DOI:** 10.3390/medicina61010115

**Published:** 2025-01-14

**Authors:** Marco Vecchiato, Nicola Borasio, Emiliano Scettri, Vanessa Franzoi, Federica Duregon, Sandro Savino, Andrea Ermolao, Daniel Neunhaeuserer

**Affiliations:** 1 Sports and Exercise Medicine Division, Department of Medicine, University of Padova, Via Giustiniani 2, 35128 Padova, Italy; marcovecchiato.md@gmail.com (M.V.); emiliano.scettri@studenti.unipd.it (E.S.); vanessa.franzoi@studenti.unipd.it (V.F.); federica.duregon@unipd.it (F.D.); andrea.ermolao@unipd.it (A.E.); daniel.neunhaeuserer@unipd.it (D.N.); 2 Institute of Mountain Emergency Medicine, EURAC Research, Viale Druso 1, 39100 Bolzano, Italy; 3 Department of Medicine, University of Padova, Via Giustiniani 2, 35128 Padova, Italy; sandro.savino@unipd.it

**Keywords:** GPS, hiking, mountain, outdoor, physical activity, safety, trail

## Abstract

*Background and Objectives*: Accurate hiking time estimate is crucial for outdoor activity planning, especially in mountainous terrains. Traditional mountain signage and online platforms provide generalized hiking time recommendations, often lacking personalization. This study aims to evaluate the variability in hiking time estimates from different methods and assess the potential of a novel algorithm, MOVE, to enhance accuracy and safety. *Materials and Methods*: A cross-sectional analysis was conducted using data from 25 Italian loop trails selected via the Wikiloc platform, considering user-uploaded GPS data from at least 20 users per trail. Real-world hiking times were compared with estimations from Komoot, Outdooractive, mountain signage, and the MOVE algorithm, which incorporates individualized biological and trail characteristics. *Results*: Significant discrepancies were observed between actual hiking times and estimates from Komoot (ΔWK: −48.92 ± 57.16 min), Outdooractive (ΔWO: −69.13 ± 58.23 min), and mountain signage (ΔWS: −29.59 ± 59.90 min; all *p* < 0.001). In contrast, MOVE showed no statistically significant difference (ΔWM: −0.27 ± 65.72 min; *p* = 0.278), providing the most accurate predictions. *Conclusions*: Current hiking time estimation methods show substantial variability and inaccuracy, which may pose safety risks. MOVE demonstrated superior accuracy, offering personalized hiking time predictions based on user-specific data and trail characteristics. Integrating such advanced tools into outdoor activity planning could enhance safety and accessibility, particularly for individuals with chronic conditions. Further studies should explore integrating real-time health data to refine these tools.

## 1. Introduction

Millions of people all over the world enjoy outdoor activities, especially in the mountain environment. In recent years, following the pandemic caused by the SARS-CoV-2, the proportion of tourists choosing the mountains as a holiday destination has increased [1,2]. Among the various outdoor activities, mountain hiking has emerged as the most popular, with well-documented benefits including enhanced physical fitness, improved mental health, and overall well-being [3,4,5,6].

Indeed, hiking at low (500–1500 m) to moderate altitudes (1500–2500 m) is a highly suitable recreational activity for both healthy individuals and for patients with non-communicable chronic diseases [7,8,9,10,11,12].

Nevertheless, there are significant safety challenges associated which frequently lead to avoidable organized search and rescue assistance. In Italy, the number of these operations has increased exponentially over the years, surpassing 10,000 annual rescue calls [13]. In 2023, about half of these rescue calls were related to accidents occurring during mountain hiking with falls and physical inability as the primary causes, accounting for approximately 46% and 26% of the cases, respectively [14].

Therefore, preventive measures should be investigated, considering outdoor activity planning as a potential strategy to address. Notably, the primary source of information about hiking trails often included consultations with local facilities and mountain signage [15,16]. Many national alpine associations also provide recommendations for people engaging in hiking. For instance, alpine organizations provide guidelines addressing physical demands, technical skills, equipment preparation, and emergency management. The Italian Alpine Club classifies mountain paths into three categories according to physical demand and technical skills required, The Alpine Association of Slovenia offers detailed safety tips and the Austrian Alpine Club emphasizes training programs and heart rate control [15,17,18]. Finally, the Swiss hiking trails association uses self-assessment tools to tailor planning advice [19]. Similarly, online platforms and web-based resources provide trail information based on generalized data with recommendations for an average hiker. In this regard, the estimated hiking time represents key information for selecting an adequate trail as well as for detailed route planning. However, hiking time estimations are generally based on trail length and elevation changes, without accounting for individual characteristics or physical condition. While some warnings and suggestions are included, fitness is rarely considered in these calculations. The generic nature of the current hiking time indications, and the fact that low physical fitness, chronic diseases, and higher cardiovascular risk can lead to heterogeneous and longer hiking times must be considered for safety reasons [11,20,21,22,23,24,25,26]. Addressing these factors could assist hikers in selecting suitable trails and implementing specific preventive measures, ultimately enhancing safety when planning hiking activities [20,25,26]. Hence, the aim of this work was to evaluate possible differences in physical and digital hiking times estimations for defined mountain trails analyzing different methodologies. A predictive model based on an individualized approach was investigated, and developed on the basis of a patented algorithm called MOVE, which is currently undergoing scientific validation. We hypothesized that a time estimation algorithm that considers not only the characteristics of the trail but also the individual characteristics of the hiker, could provide a more accurate estimate of the time required to complete a mountain hike.

## 2. Materials and Methods

This cross-sectional research project was carried out at the Division of Sports and Exercise Medicine of the Department of Medicine, University of Padova, using a quantitative approach to compare hiking times estimated from various sources with travel hiking times monitored and uploaded by users of the Wikiloc platform [27]. Since the study used publicly available, anonymized data, ethical approval was not required, in accordance with institutional and national research guidelines [28].

### 2.1. Trail Selection and Eligibility Criteria

Wikiloc is a digital freemium platform that serves as a repository and sharing service for outdoor activities and trails across the globe where registered members upload their GPS trails and waypoints. The Wikiloc platform was used for the selection of the trail routes and for obtaining real hiking times.

Twenty-five Italian trails, were chosen and analyzed with a standardized research method:

- Only the hiking activity was considered.
- The tolerated variability in performing the respective hiking trail was ±1 km.
- Trails with at least 20 uploaded users were selected.
- Only circular trails with the same direction of travel were selected.

The first 20 trails meeting the inclusion criteria were selected (search concluded on 3 April 2024). The original uploaded data set of trail characteristics was analyzed in order to obtain information regarding the total time spent to complete the trail, time in motion, GPS coordinates, distance, elevation gain, reported technical difficulty level on a 3-point scale (1 = easy; 2 = moderate; 3 = difficult), the most popular month of the year and sex of the user.

### 2.2. Hiking Time Estimation Methods

The real-world hiking times have been uploaded by users to the Wikiloc platform and could be compared with four hiking estimation methods:

- Komoot. Komoot is a web platform with different functionalities related to outdoor activities (route planner, navigation app, tour directory, and social network). It offers a generic time estimation in which the only customizable variable is the training level of the user. Not knowing the physical activity level of the selected users, intermediate training level (default setting) was considered for all users [29].
- Outdooractive. Outdooractive is another well-known web platform with different functionalities for outdoor physical activities (route planner, navigation app, and tour directory). The “Route planner” function allows an estimation of the hiking time for a specific uploaded track. No details about the individual user are required [30].
- Mountain signage. The hiking times of the different routes as provided by the mountain physical signposts of the Italian Alpine Club (CAI) have been identified through official online sources. The CAI suggests for an average trained hiker a positive elevation gain of 350 m and a negative elevation gain of 500 m in one hour of hiking. For flat sections, the pace considered is 3.5–4 km/h. Moreover, if the trail is at altitudes above 2800–3000 m, a positive elevation gain of 250 m and a negative elevation gain of 400 m in one hour of hiking are predicted. For estimated hiking times longer than four hours, adjustments are made to the next half-hour or hour [15].
- MOVE. MOVE is a scientifically developed digital algorithm, with an Italian national patent (deposit number: 102021000026513; approval 24.01.2024). It aims to estimate the expected hiking time needed to complete a hiking route, based on certain biological parameters of the user and physical characteristics of the trail, including altitude. More specifically, MOVE calculates maximal oxygen consumption (VO_2_ max) using the Whaley et al. equation, which considers factors such as sex, age, physical activity level, and body mass index (BMI). For the purposes of this study, a BMI of 22 kg/m^2^ was utilized as the standard for all subjects, thereby assuming a normal weight classification [31]. Additionally, cardiovascular risk is assessed using the ESC SCORE 2 chart, incorporating cholesterol levels, blood pressure, and smoking habits. Walking oxygen consumption is scaled progressively based on cardiovascular risk, ranging from 40% of VO_2_ max for very high-risk individuals to 69% of VO_2_ max for low-risk individuals [32]. For the prediction of the walking speed, the system employs the walking equation, which accounts for both uphill (positive grade) and downhill (negative grade) conditions [33].

To ensure realistic outputs, the predicted walking speed is constrained between 0.27 m/s and 1.75 m/s, providing personalized hiking time recommendations tailored to moderate-intensity effort levels. This approach aligns with the user’s cardiovascular fitness and safety requirements [34]. For this study, the only biological parameter available was the sex of the users. An intermediate training level was chosen in order to ensure consistency with the Komoot tool assumptions and to align with the findings of a previous study, which indicated that the majority of individuals engaging in hiking reported a moderate level of physical activity [35]. Additionally, data from the Wikiloc platform predominantly represent a subpopulation with a moderate-to-high level of physical activity. This combination of factors supports the assumption of an intermediate training level. Since 50% of the hikers in Italy are between 30 and 50 years old, an average age of 40 was considered [36].

### 2.3. Statistical Analyses

Qualitative variables were expressed as frequency (percentage) and quantitative variables as mean and standard deviation or median (interquartile range). The differences between the real-time uploads on Wikiloc and the hiking times estimated by Komoot, Outdooractive, mountain signage, and the MOVE algorithm were expressed as deltas (ΔWK, ΔWO, ΔWS, ΔWM, respectively) in minutes. The delta values are reported with a 95% confidence interval. Paired Wilcoxon tests were employed to compare estimates with the different methods and real hiking times due to the non-normal distribution of the data, as confirmed by the Shapiro–Wilk test. Subgroup analyses were conducted to assess variations according to gender (male vs. female), trail difficulty (easy vs. moderate vs. difficult), length (<10 km vs. ≥10 km), and elevation gain (<600 m vs. ≥600 m), as these factors are expected to influence hiking performance. To compare these subgroup samples, the Mann–Whitney U test for independent samples was used. A *p* < 0.05 was considered as a statistically significant difference. Data analyses were conducted with SPSS version 26.0.

## 3. Results

**Figure 1:**
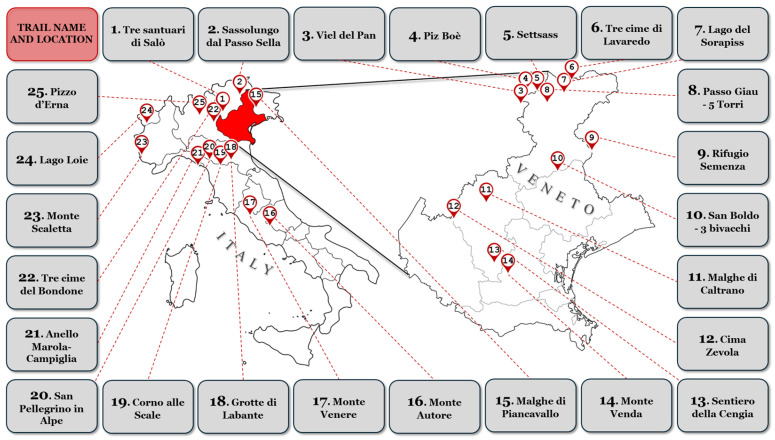
The figure illustrates the geographical distribution of 25 selected hiking trails across Italy that meet the study’s inclusion criteria. The trails are numbered and labeled with their respective names and locations. The map highlights the Veneto region in red, with several trails concentrated in this area, while others are distributed across various regions of northern and central Italy. Each trail is connected to its respective location on the map via dashed red lines, with the trail names listed in corresponding boxes.

The 25 trails identified through the Wikiloc platform are shown in Figure 1 for a total of 500 different user analyses (72% male). More details of each individual trail are represented in Figure S1 and Table S1 in the Supplementary Materials. The average distance hiked was 10.88 km (5.38–17.18 km). The overall maximum and minimum altitudes were 1817.37 m and 1314.44 m, respectively, with a mean positive elevation difference of 629.36 m. The technical difficulty most frequently reported by users was moderate (*n* = 396, 79%). The most popular month to hike was August (*n* = 124; 25%). The main user and trail information of the 25 selected routes are described in Table 1.

**Figure 2:**
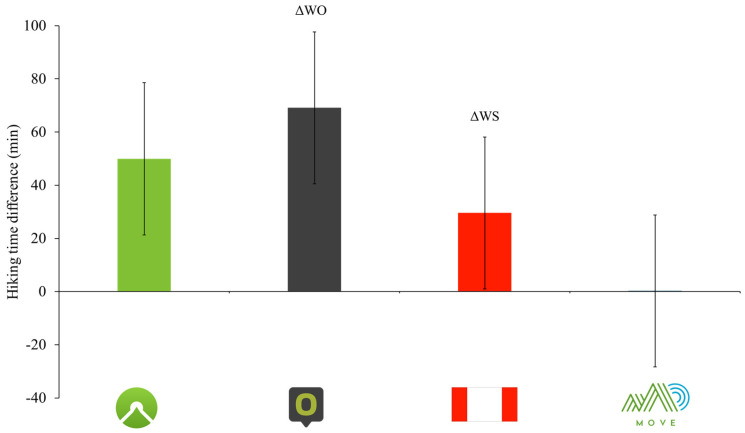
The figure displays the mean differences in hiking time estimates (in minutes) between actual hiking times and those provided by four methods: Komoot (ΔWK, green bar), Outdooractive (ΔWO, black bar), mountain signage (ΔWS, red bar), and MOVE (ΔWM, blue bar). Error bars represent the standard deviation of the differences. The results indicate that Komoot, Outdooractive, and mountain signage consistently overestimate hiking times, whereas MOVE shows minimal deviation from actual times, demonstrating higher accuracy.

The difference between user-reported measured times and hiking times estimated by included calculation methods in the whole sample and in the subgroups are represented in Table 2 and Figure 2. Complete data for all trails are provided in Supplementary Material, Table S2. Statistically significant differences between these real hiking times and those estimated by Komoot, Outdooractive, and the mountain signage were observed with a mean difference of ΔWK −48.92 ± 57.16 min [95% CI: −54.09; 44.04], ΔWO −69.13 ± 58.23 min [95% CI: −74.24; 64.01] and ΔWS −29.59 ± 59.90 min [95% CI: −34.77; 24.41], respectively (all *p* < 0.001). No statistically significant difference between real hiking times and those predicted by MOVE has been observed (ΔWM: *p* = 0.278; Figure 2), showing an average difference of −0.27 ± 65.72 min [95% CI: −6.17; 5.40] from actual hiking times. Moreover, MOVE reported a non-significant difference between estimated and real hiking times in the largest number of trails (14 trails out of 25) which was superior when compared with the other hiking time estimation methods (Komoot: 5; Outdooractive, 0; Mountain signage: 8).

**Figure 3:**
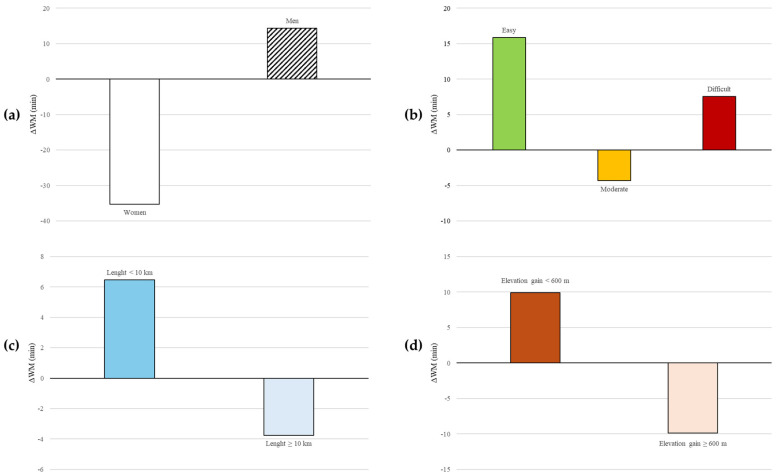
Differences in MOVE’s hiking time estimates (ΔWM, in minutes) across various subgroup analyses. MOVE underestimates hiking times for women while slightly overestimating for men (*p* < 0.001) (**a**). Low and high difficulty trails showing positive deviations while average difficulty trails being nearly accurate (*p* = 0.060) (**b**). Trails shorter than 10 km show positive mild overestimation, while those longer than or equal to 10 km exhibit slight underestimation (*p* = 0.229) (**c**). Trails with elevation gain below 600 m show overestimated hiking times, whereas trails with elevation gain equal to or greater than 600 m are underestimated (*p* < 0.001) (**d**).

MOVE showed significant differences in estimated versus real reported hiking times when the sample was grouped by sex and elevation gain (both *p* < 0.001) but not when analyzed by trail difficulty (*p* = 0.060) or trail length (*p* = 0.229; Table 2 and Figure 3).

## 4. Discussion

### 4.1. Incorrect Hiking Time Estimation as Potential Risk Factor

This study represents the first investigation into possible discrepancies between actual hiking times on mountain trails and hiking times estimated by different widespread and commonly used digital and physical methods for outdoor activity planning. A meaningful difference and heterogeneity are particularly concerning given the growing diversity within the hiking population, which includes not only healthy individuals but also elderly people and those affected by chronic conditions such as cardiovascular diseases, diabetes, metabolic syndrome, or respiratory disorders [9,10,23,37,38]. Hiking is a popular outdoor activity but requires careful planning, particularly when it comes to estimating the time needed to complete a certain trail. Accurate time estimation is crucial not only for ensuring that hikers can complete their excursion safely and within daylight hours but also for managing their energy and resources efficiently [25,35].

Our research shows that the available estimation methods present a great variability and a significant difference from the actual hiking times, which appear to be better predicted by MOVE, an algorithm specifically developed for this purpose. Inaccuracy in hiking times predictions can have profound implications on hikers’ safety, which may result in a misperception of the true level of difficulty and the potential risks associated with hiking. This can lead to a reduction in the level of preparation or caution, increasing the likelihood of exposure to adverse conditions and the potential for accidents [20,35,39,40]. In mountain environments, where weather conditions can change rapidly and trails can vary greatly in difficulty, accurate time planning is crucial, especially considering that delays are frequent causes of rescue calls [41]. In addition, it should be considered that the busiest month for hiking is August and that the ambient temperature can also affect the duration of hikes, thus increasing the risk of heat-related illnesses during strenuous activities [42,43].

### 4.2. Why Different Hiking Time Estimates?

The best-known hiking time estimation method is offered by vertical mountain signage which from our study seems to slightly overestimate the real times uploaded by users. There are several valuable international classifications and signage systems for mountain trails, such as the Swiss or Italian classifications, which are continuously being updated [15,16]. Signage on mountain trails indicates expected hiking times, which are calculated for an average person and do not consider individual characteristics affecting exercise capacity and cardiovascular risk [32]. Indeed, individuals with chronic diseases may face different risks when hiking in mountain environments, especially at high altitudes [11,20,21,22]. Psychophysical stress demands combined with hypoxia and temperature modifications can increase the risk of cardiovascular events and significantly affect performance [11,44]. In this context, online platforms or digital tools could play a crucial role not only in promoting the tourism of mountain areas but also in providing updated and detailed information about the trails. On the other hand, the increased visibility through these platforms may also lead to a greater influx of inexperienced hikers in mountain areas [45]. Furthermore, the lack of clear information regarding the physical ability required to complete a hike creates uncertainty, which could become a risk factor or influence their decision to undertake an excursion [35,40].

It has already been demonstrated that there is a need for a sufficient level of physical activity and information about related physical and psychological risks in order to safely participate in a hike [35,40,46]. Both Komoot and Outdooractive overestimate the actual hiking time of users, with the former taking into account the user’s physical activity level. MOVE is based on an algorithm that utilizes a wide range of person-specific data, including anthropometric parameters, gender, age, physical activity level, cardiovascular risk factors (such as smoking history, blood pressure, and cholesterol), and the presence of chronic diseases to predict individual hiking time. The algorithm could also consider the weight of the backpack, which may result in different energy requirements to complete a trail [25,47]. This information is then integrated with the GPS track of the hiking route, thus enabling the calculation of the length, slope and elevation of the trail, allowing for the provision of personalized hiking advice and recommendations, which are particularly based on subjects’ physical fitness. Moreover, since maximal oxygen consumption decreases by approximately 1% per 100 m above 1500 m, and the relative difficulty of hiking progressively increases with elevation, this must also be considered in such predictions [44]. The provided MOVE outputs include tailored hiking time recommendations, estimated energy expenditure, and specific health-related precautionary measures, particularly useful for individuals with chronic conditions, thereby promoting safety by adequately planning this outdoor activity. This tool should enable users to assess the suitability of a chosen itinerary based on their individual physical characteristics and health status, ensuring a safer and more informed approach to outdoor recreational physical activities. Furthermore, our study confirms the findings of Coetzee et al., suggesting a significant inter-individual variability in hiking times for the same route [35]. Methodologies that fail to take into account the individual characteristics of the subjects or that solely consider the level of physical activity seems inadequate for providing indications of hiking time that are practically useful for planning purposes. A method that accurately considers individual characteristics would appear to provide a more realistic estimate of the required individual performance.

### 4.3. How This Tool Can Impact Prevention in the Mountain Environment

Digital tools such as MOVE have the potential to serve as valuable educational instruments, providing guidance, for example, on the importance of sun protection, technical clothing, and the proper use of equipment such as backpacks and trekking poles [48,49,50]. The use of trekking poles has been shown to significantly reduce muscle fatigue, perceived exertion levels, and the risk of muscle injuries [49,50]. However, this study did not procure data regarding the use of trekking poles, and therefore, it was not possible to analyze differences in hiking times between users who utilized trekking poles and those who did not. Additionally, predicting the availability of natural light at the expected time of arrival can serve as a critical preventive measure [26,51]. In mountain environments, where conditions can change rapidly, hikers relying on inaccurate outdoor activity planning may find themselves unprepared for the terrain or weather conditions they encounter [43,52,53]. This is particularly dangerous for individuals with chronic conditions, such as cardiovascular, pulmonary diseases, or diabetes, who may require more precise hiking time estimates to manage their health issues effectively [10,21,23]. The algorithm can adjust its estimates based on the presence of specific health conditions, that could significantly impact performance. For elderly subjects or people with chronic diseases, it calculates lower walking oxygen consumption and consequently longer estimated times. It also offers general and specific advice and precautions based on the latest scientific evidence [11,21,32,54]. These features are designed to support both healthy users and those with pre-existing conditions. It is important to note that the algorithm is intended for individuals with stable conditions and good therapeutic control. Moreover, it is strongly recommended that individuals with health problems consult their physician before participating in such outdoor activity [21].

In recent years, the implementation of preventive measures in mountain environments has assumed great relevance and importance because of the significant increase in the number of accidents that have occurred [13,55]. The role of technology in improving the safety of mountain hiking cannot be overstated. The use of drones, telemedicine, and localization devices in search and rescue operations has already begun to be tested in order to reduce response times and improve outcomes in rescue scenarios [2,56]. Furthermore, as suggested by Mieda et al., technologies could also be integrated into hiking apps and wearable devices, which are more and more accessible to the public, aiming to provide recommendations and real-time self-monitoring to help prevent such adverse events [26].

The MOVE algorithm, which considers individual biological parameters and physical characteristics of the trail, may represent a significant advancement in this context, offering personalized predictions that could lead to increased awareness and safety for hikers and promote the health-related benefits of hiking in a natural environment.

### 4.4. Limitations and Future Perspectives

Despite the high number of users analyzed, this study has some limitations arising from the lack of real information about biological parameters like age and BMI. Additionally, some possible pathological conditions, limb length discrepancies, or gait parameters, the influence of backpacks and the presence or absence of hiking companions during the excursions may have influenced the hiking times. Despite the majority of hikes taking place in August, there was considerable variability in the seasons of the hikes for which data were available. Furthermore, the absence of data on weather conditions and terrain type could introduce additional biases that were not considered in this study. The approximations made in this study represent an attempt to standardize and mitigate potential estimation errors but cannot adequately consider the biological variability between users. A further inherent limitation of this study concerns the selection of the sample from the Wikiloc platform dedicated to sharing hikes within a community. It can be assumed that the sample obtained is constituted of a sub-population characterized by a generally high physical activity level, substantial experience in outdoor activities, and a broad knowledge of the mountain environment. Future research should focus on refining these outdoor planning tools also based on individual health data, potentially integrated with wearable technology to provide real-time adjustments based on the hiker’s current physical status.

## 5. Conclusions

A large variability exists among methods used for estimating hiking times and trail planning. The MOVE algorithm proved to be the estimation method that best approximates real-world hiking times. Thus, the integration of more accurate and personalized trail time estimation tools into hiking planning platforms could play a critical role in enhancing safety. Moreover, the development of educational programs for hikers regarding the associated risks, the importance of outdoor activity planning and essential precautionary measures, could further increase safety, particularly for those subjects with chronic conditions, who may thereby be able to experience the health benefits of physical activity in green environments.

## Figures and Tables

**Table 1:** Main data of the selected trails. Quantitative variables are represented as means and standard deviations. Qualitative data are expressed as frequency (percentage).

Trail	Gender (Men; %)	Length (km)	Average Elevation Gain (m)	Technical Difficulty (Easy/Moderate/Difficult)	Most Popular Month	Average Max Altitude (m)	Average Min Altitude (m)	Average Coordinates	Hiking Time (h:min)
Trail #1	12 (60%)	9.92 ± 0.65	511 ± 43	4/15/1	March	534 ± 20	130 ± 35	1770 ± 578	2:39 ± 39
Trail #2	15 (75%)	11.49 ± 0.39	791 ± 107	5/14/1	August	2673 ± 19	2059 ± 19	2008 ± 81	3:29 ± 37
Trail #3	8 (40%)	13.82 ± 0.87	570 ± 70	1/18/1	August	2492 ± 36	2094 ± 24	2368 ± 252	3:36 ± 44
Trail #4	12 (60%)	9.95 ± 0.52	1003 ± 105	2/16/2	August	3127 ± 23	2235 ± 18	1728 ± 159	3:23 ± 61
Trail #5	16 (80%)	11.36 ± 0.39	555 ± 45	1/16/3	August	2320 ± 15	2062 ± 13	2022 ± 68	3:31 ± 21
Trail #6	17 (85%)	10.49 ± 0.37	438 ± 31	2/17/1	July	2468 ± 26	2188 ± 28	1921 ± 264	3:03 ± 24
Trail #7	13 (60%)	13.70 ± 0.49	859 ± 144	0/19/1	August	2298 ± 14	1731 ± 13	2363 ± 554	3:58 ± 58
Trail #8	14 (60%)	11.38 ± 0.30	702 ± 67	0/20/0	August	2569 ± 21	2124 ± 18	2014 ± 93	3:43 ± 42
Trail #9	15 (60%)	11.04 ± 0.38	850 ± 51	2/16/2	October	2025 ± 35	1209 ± 21	1921 ± 107	3:20 ± 38
Trail #10	13 (60%)	10.92 ± 0.83	628 ± 70	5/15/0	March	1274 ± 44	699 ± 21	1833 ± 202	2:47 ± 38
Trail #11	11 (60%)	13.12 ± 1.25	284 ± 43	2/15/3	August	1418 ± 32	1253 ± 26	2068 ± 328	3:12 ± 67
Trail #12	14 (60%)	10.25 ± 1.19	751 ± 109	1/19/0	July	1921 ± 62	1232 ± 48	1777 ± 233	3:02 ± 37
Trail #13	17 (60%)	10.25 ± 0.47	497 ± 20	4/16/0	November	425 ± 24	49 ± 15	1793 ± 110	2:23 ± 24
Trail #14	13 (60%)	8.38 ± 0.43	374 ± 52	1/14/5	March, June, July	594 ± 65	331 ± 60	1395 ± 157	1:55 ± 29
Trail #15	19 (60%)	8.81 ± 0.63	223 ± 15	0/13/7	August	1371 ± 15	1163 ± 11	1523 ± 163	2:09 ± 18
Trail #16	16 (60%)	9.09 ± 0.53	379 ± 32	9/11/0	October, November	1854 ± 24	1558 ± 28	1567 ± 106	2:33 ± 33
Trail #17	13 (60%)	5.38 ± 0.65	265 ± 18	4/16/0	August, September, October	848 ± 38	586 ± 37	907 ± 104	1:40 ± 58
Trail #18	15 (60%)	9.23 ± 0.56	383 ± 45	4/16/0	June, September, November	746 ± 29	449 ± 13	1613 ± 194	2:20 ± 31
Trail #19	12 (60%)	14.32 ± 1.04	899 ± 87	4/16/0	July	1940 ± 19	1295 ± 84	2418 ± 227	4:39 ± 84
Trail #20	15 (60%)	14.01 ± 0.77	433 ± 61	4/16/0	August	1674 ± 23	1382 ± 24	2354 ± 186	3:29 ± 36
Trail #21	12 (60%)	8.62 ± 0.64	689 ± 128	3/16/1	March	595 ± 89	101 ± 88	1509 ± 120	3:07 ± 71
Trail #22	14 (60%)	10.67 ± 0.57	788 ± 65	0/13/7	June	2235 ± 302	1620 ± 304	1791 ± 219	3:29 ± 63
Trail #23	16 (60%)	13.90 ± 1.31	1165 ± 103	1/15/4	July	2840 ± 40	1845 ± 73	2387 ± 261	4:11 ± 44
Trail #24	14 (60%)	12.09 ± 0.58	827 ± 35	3/17/0	July	2428 ± 214	1658 ± 218	2134 ± 154	3:37 ± 41
Trail #25	16 (60%)	10.24 ± 1.15	857 ± 83	1/18/1	August	1341 ± 50	558 ± 32	1722 ± 326	2:53 ± 65
TOTAL	352 (70%)	10.90 ± 2.22	628 ± 255	63/397/40	August (25%)	1760 ± 793	1264 ± 709	1876 ± 476	3:07 ± 62

**Table 2:** Comparison of real uploaded hiking times (Wikiloc) and selected estimation methods between subgroups and in the whole sample.

Trail	Wikiloc (hh:mm)	Komoot (hh:mm)	∆WK (min)	*p*	Outdoor Active (hh:mm)	∆WO (min)	*p*	Mountain Signs (hh:mm)	∆WS (min)	*p*	MOVE (hh:mm)	∆WM (min)	*p*
Men (*n* = 352)	3:11 ± 1:03	3:57 ± 1:06	−45.92 ± 57.01	0.183	4:16 ± 1:07	−65.09 ± 58.32	0.082	3:38 ± 1:03	−26.65 ± 59.50	0.161	2:57 ± 1:05	14.37 ± 61.82	<0.001
Women (*n* = 141)	2:55 ± 58	3:48 ± 1:05	−53.48 ± 56.73		4:10 ± 59	−75.08 ± 55.33		3:29 ± 1:00	−34.99 ± 57.36		3:30 ± 1:14	−35.24 ± 62.96	
Easy (*n* = 63)	2:58 ± 1:00	3:24 ± 59	−25.67 ± 58.81	<0.001	3:46 ± 1:04	−47.86 ± 61.63	0.005	3:04 ± 55	−5.47 ± 62.04	0.001	2:43 ± 1:00	15.86 ± 63.57	0.060
Moderate (*n* = 396)	3:04 ± 59	3:56 ± 1:05	−51.63 ± 54.77		4:16 ± 1:05	−71.95 ± 56.27		3:36 ± 1:02	−32.64 ± 56.99		3:08 ± 1:09	−4.31 ± 63.40	
Difficult (*n* = 40)	3:43 ± 1:01	4:47 ± 1:05	−64.73 ± 57.21		5:02 ± 55	−79.45 ± 58.36		4:25 ± 56	−42.70 ± 54.60		3:35 ± 1:13	7.58 ± 74.81	
Length <10 km (*n* = 171)	2:25 ± 52	2:54 ± 49	−28.63 ± 52.92	<0.001	3:19 ± 52	−54.56 ± 55.07	<0.001	2:49 ± 59	−24.06 ± 60.91	0.131	2:18 ± 50	6.47 ± 54.14	0.229
Length ≥10 km (*n* = 329)	3:29 ± 55	4:28 ± 50	−59.47 ± 56.51		4:46 ± 52	−76.69 ± 58.46		4:01 ± 50	−32.47 ± 57.70		3:33 ± 1:04	−3.77 ± 70.81	
Gain <600 m (*n* = 243)	2:39 ± 50	3:07 ± 45	−27.63 ± 40.22	<0.001	3:28 ± 48	−48.37 ± 48.73	<0.001	2:49 ± 42	−9.74 ± 46.38	<0.001	2:29 ± 46	9.89 ± 45.61	<0.001
Gain ≥600 m (*n* = 257)	3:33 ± 1:00	4:42 ± 49	−69.05 ± 63.26		5:02 ± 45	−88.76 ± 59.73		4:22 ± 44	−48.27 ± 63.24		3:43 ± 1:08	−9.88 ± 79.12	
Whole sample (*n* = 500)	3:07 ± 1:02	3:56 ± 1:07	−48.92 ± 57.16	<0.001	4:16 ± 1:06	−69.13 ± 58.23	<0.001	3:37 ± 1:03	−29.59 ± 59.90	<0.001	3:07 ± 1:09	−0.27 ± 65.72	0.278

## Data Availability

The raw data supporting the conclusions of this article will be made available by the authors on request.

## Supplementary Materials

The following supporting information can be downloaded at: https://www.mdpi.com/article/10.3390/medicina61010115/s1, Figure S1: Trail name, Italian region, distance, elevation gain, and map of the 25 trails selected by Wikiloc.; Table S1: Wikiloc hyperlinks to sources used for the study divided by trail.
