# Supplementary material for: Are Suggested Hiking Times Accurate? A Validation of Hiking Time Estimations for Preventive Measures in Mountains
Source: Medicina (Kaunas). 2025 Jan 14;61(1):115. doi: 10.3390/medicina61010115 (PMC11766859; doi:10.3390/medicina61010115)
Supplement: Supplementary file 1 [file medicina-61-00115-s001.zip › medicina-3386819-supplementary.pdf]

Figure S1. Trail name, Italian region, distance, elevation gain and map of the 25 trails selected by Wikiloc.

1. **Tre santuari di Salò**

Lombardy (Brescia)

9.9 km

511 m

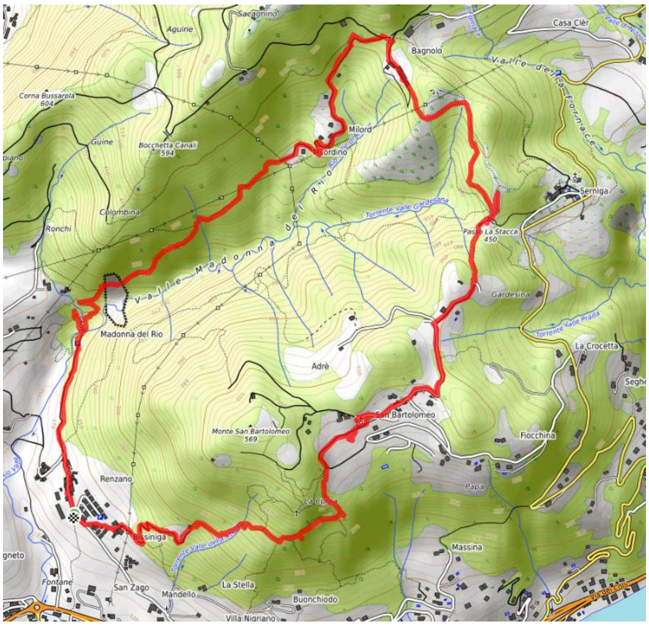

2. **Sassolungo dal Passo Sella**

Trentino-Alto Adige (Bolzano)

11.5 km

791 m

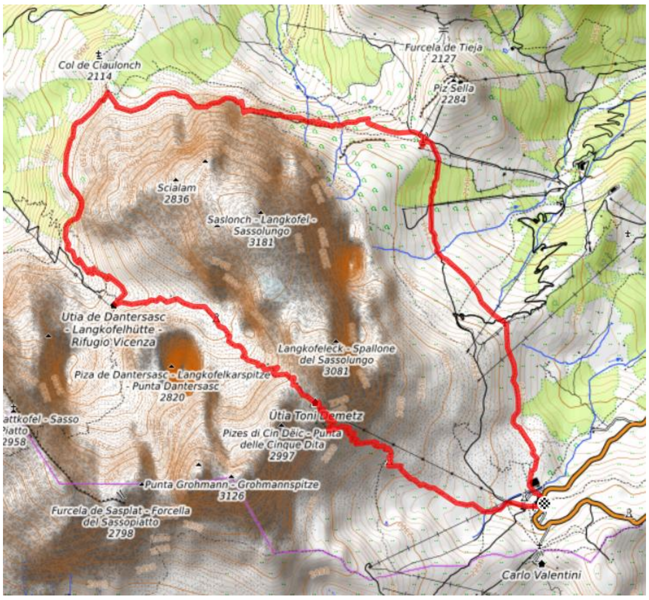

3. Viel del Pan

Trentino-Alto Adige (Trento), Veneto (Belluno)

13.8 km

570 m

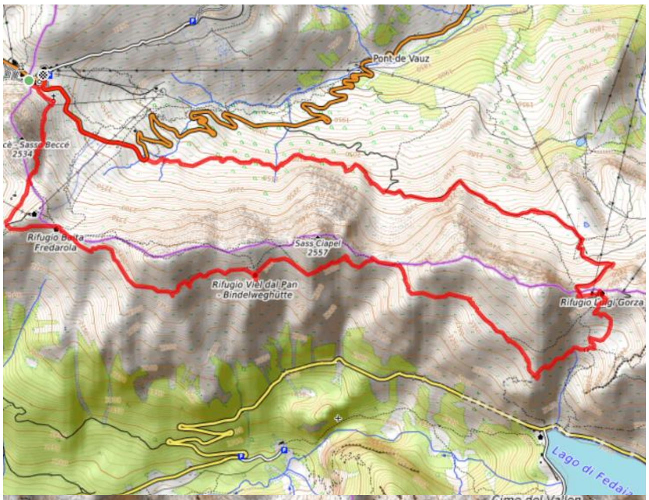

4. Piz Boè

Trentino-Alto Adige (Trento), Veneto (Belluno)

10.0 km

1003 m

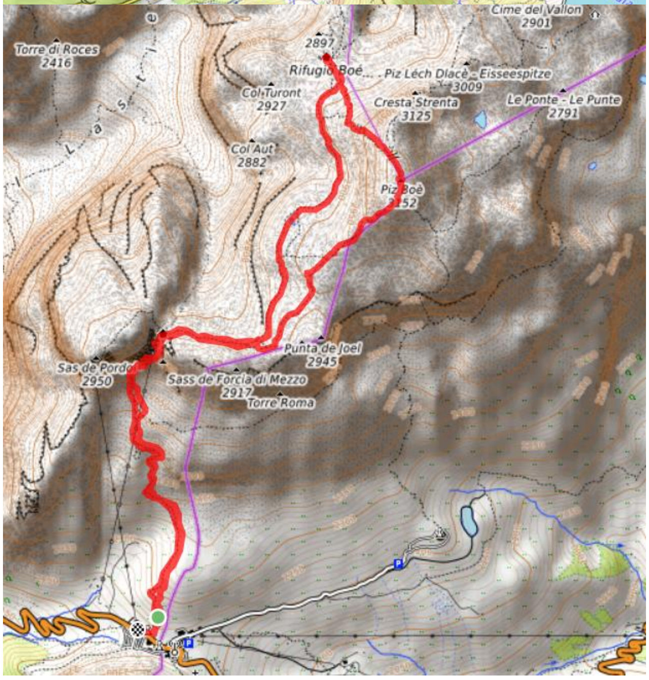

5. Settsass

Veneto (Belluno)

11.4 km

555 m

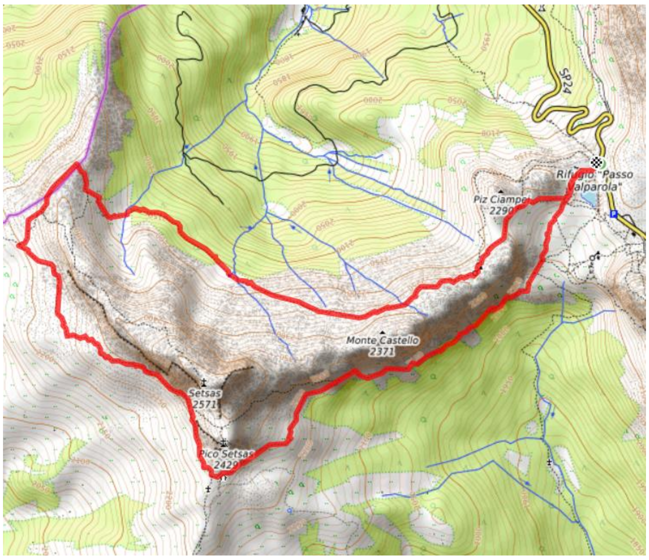

6. **Tre Cime di Lavaredo**

Veneto (Belluno), Trentino-Alto Adige (Bolzano)

10.5 km

438 m

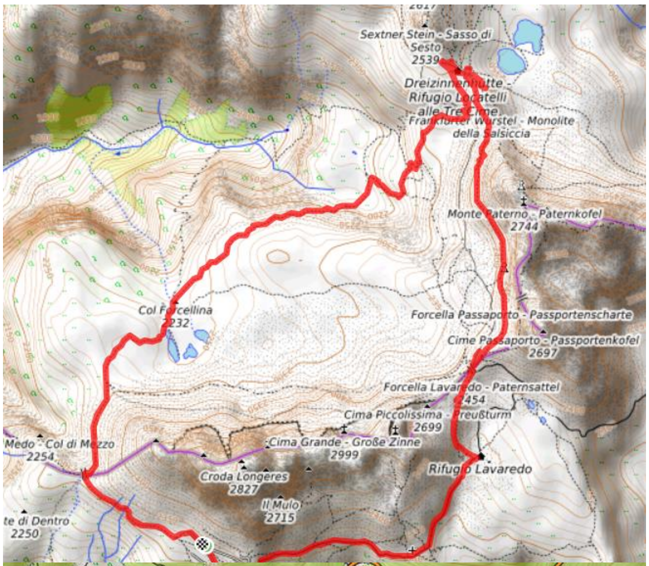

7. **Lago del Sorapiss**

Veneto (Belluno)

13.7 km

859 m

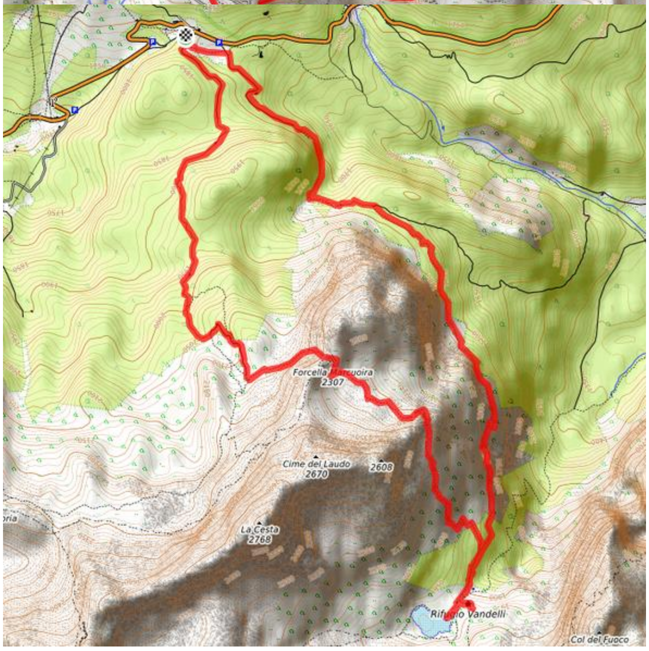

8. **Passo Giau – 5 Torri**

Veneto (Belluno)

11.4 km

702 m

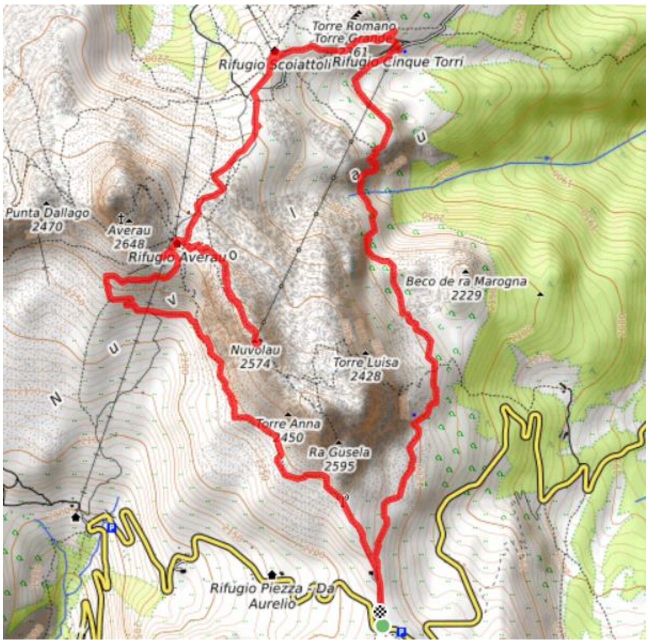

9. Rifugio Semenza

Veneto (Belluno)

11.0 km

850 m

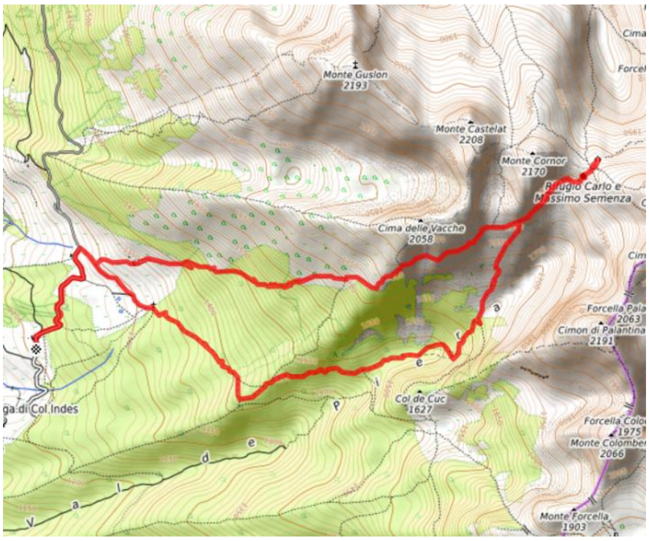

10. San Boldo – 3 bivacchi

Veneto (Treviso, Belluno)

10.9 km

628 m

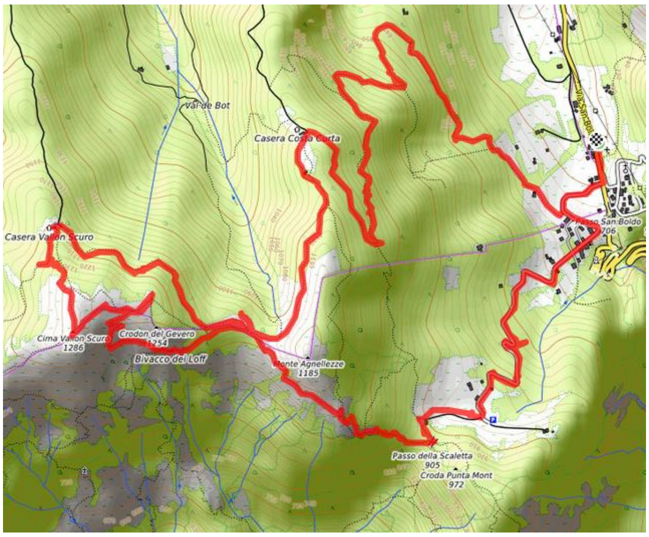

11. Malghe di Caltrano

Veneto (Vicenza)

13.1 km

284 m

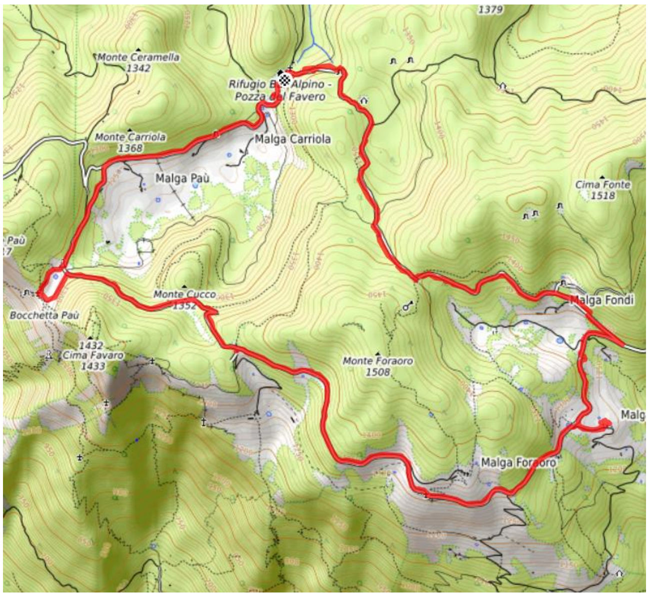

12. Monte Zevola

Veneto (Vicenza, Verona)

10.3 km

751 m

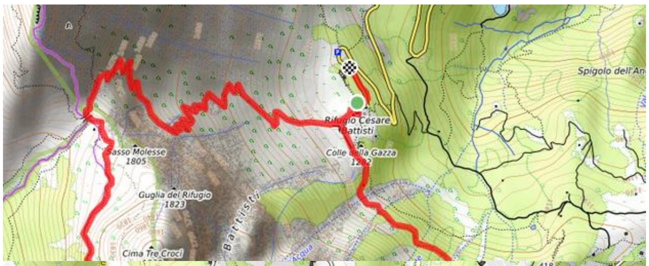

13. Sentiero della Cengia

Veneto (Vicenza)

10.3 km

497 m

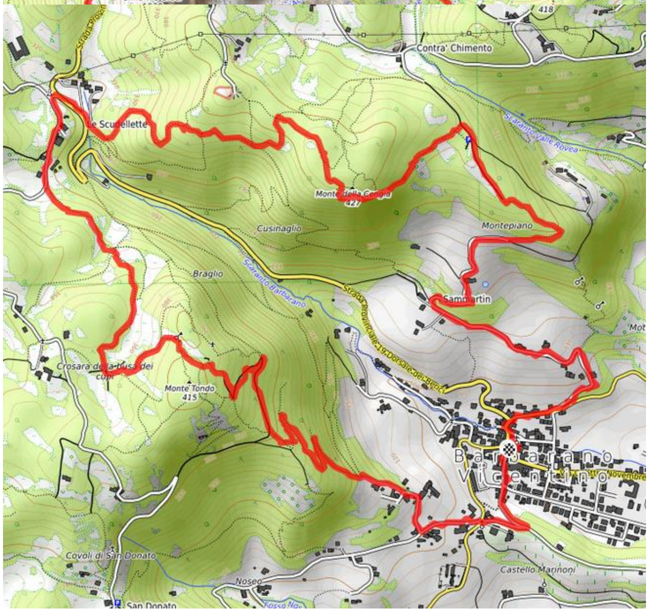

14. Monte Venda

Veneto (Padova)

8.4 km

374 m

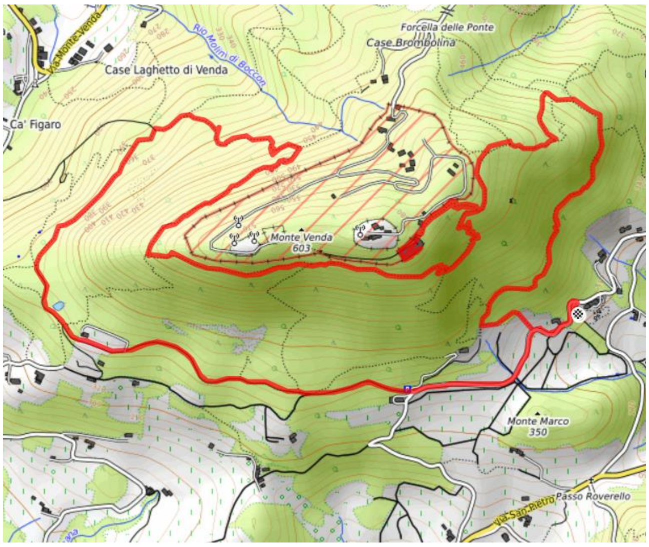

15. Malghe di Piancavallo

Friuli-Venezia Giulia (Pordenone)

8.8 km

223 m

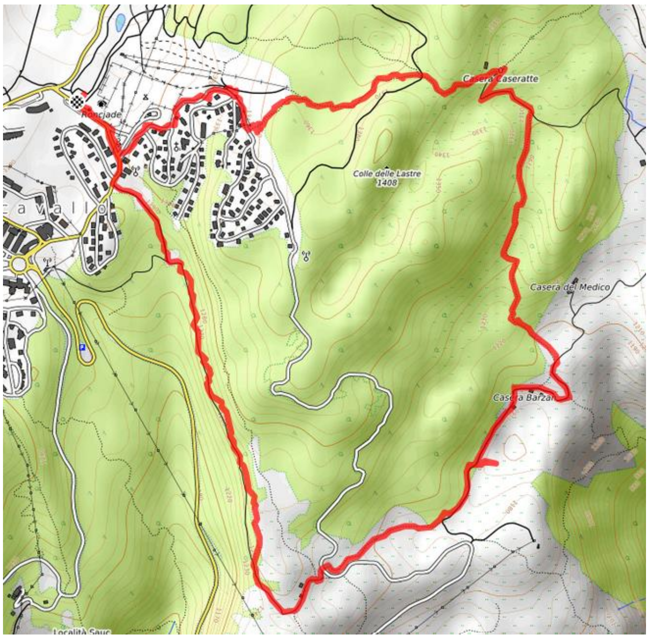

16. Monte Autore

Lazio (Rome)

9.1 km

379 m

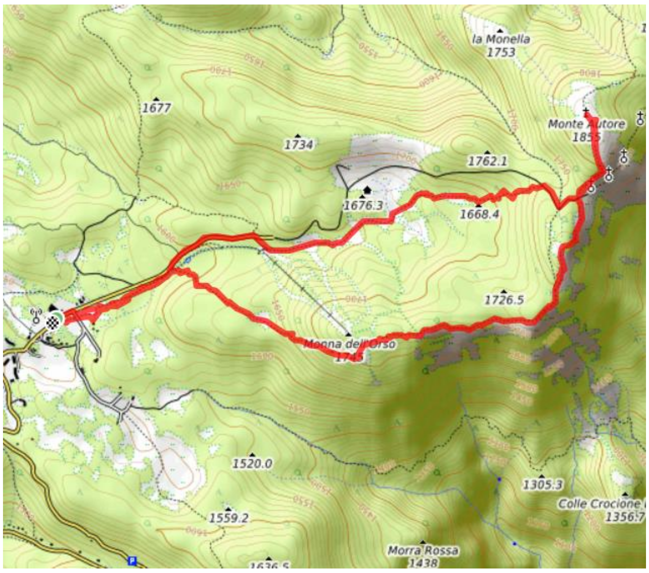

17. Monte Venere

Lazio (Viterbo)

5.4 km

265 m

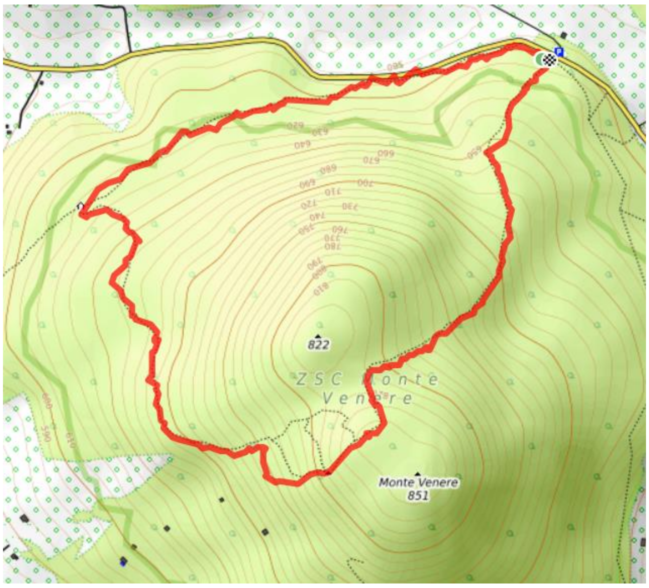

18. Grotte di Labante

Emilia-Romagna (Bologna)

9.2 km

383 m

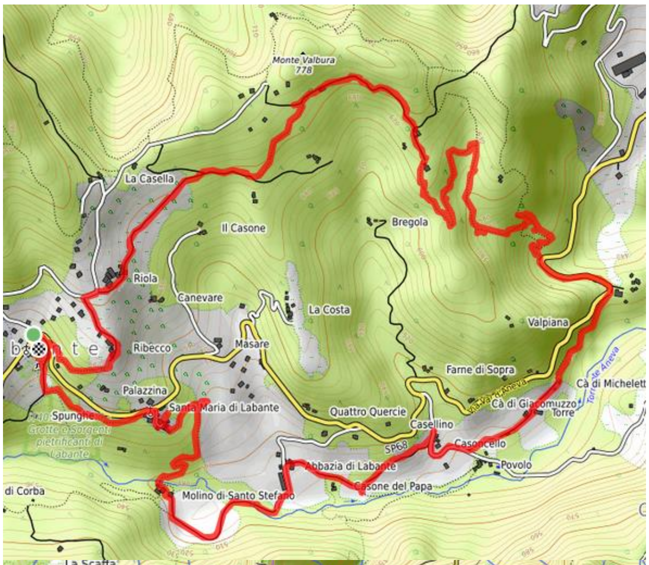

19. Corno alle Scale

Emilia-Romagna (Bologna), Tuscany (Pistoia)

14.3 km

899 m

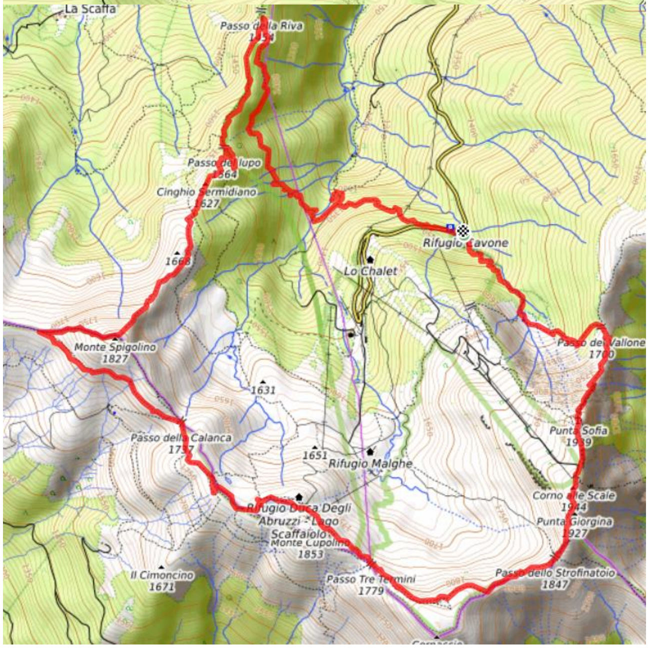

20. San Pellegrino in Alpe

Tuscany (Lucca)

14.0 km

433 m

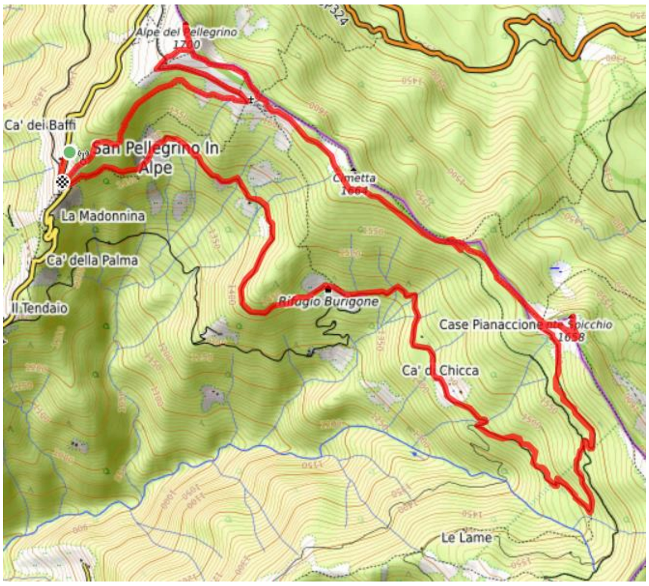

21. Anello Marola – Campiglia

Liguria (La Spezia)

8.6 km

689 m

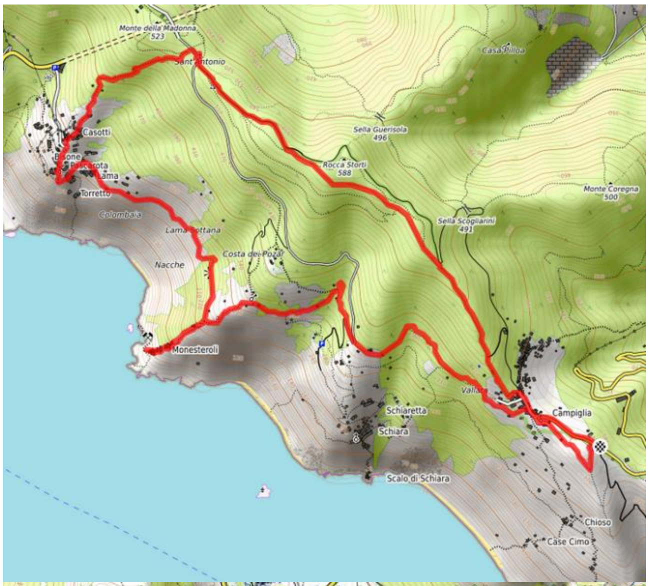

22. Tre Cime del Bondone

Trentino-Alto Adige (Trento)

10.7 km

788 m

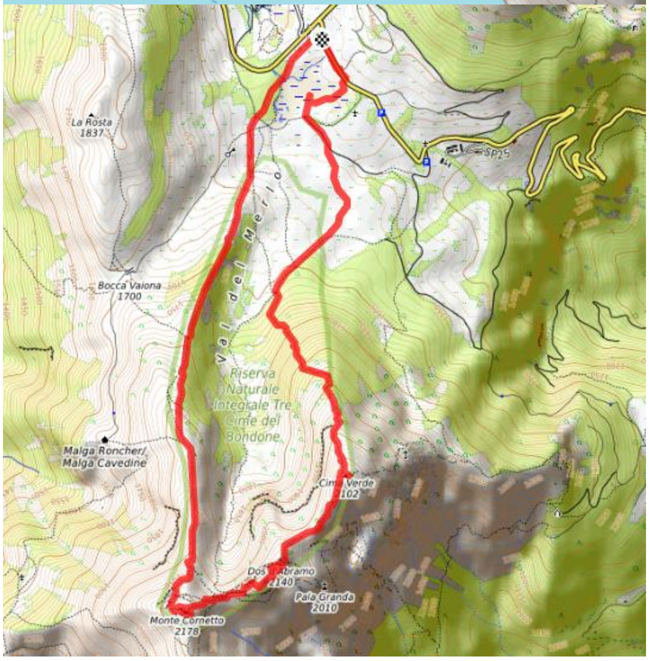

23. Monte Scaletta

Piedmont (Cuneo)

13.9 km

1165 m

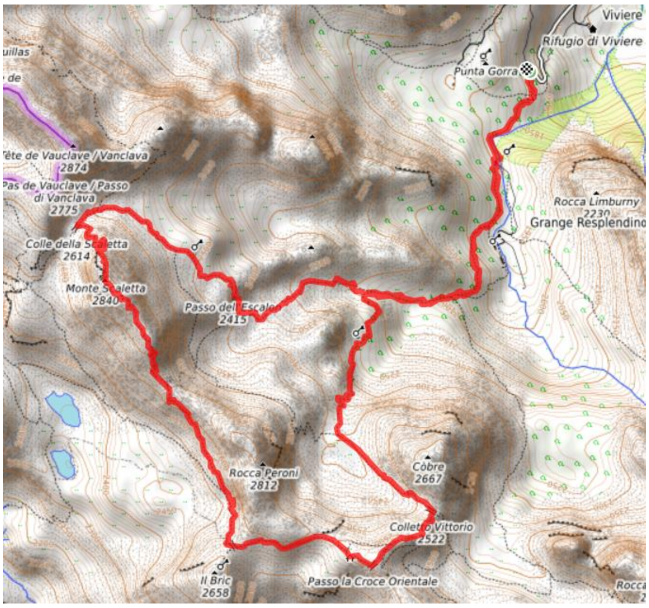

24. Lago Loie

Aosta Valley (Aosta)

12.1 km

827 m

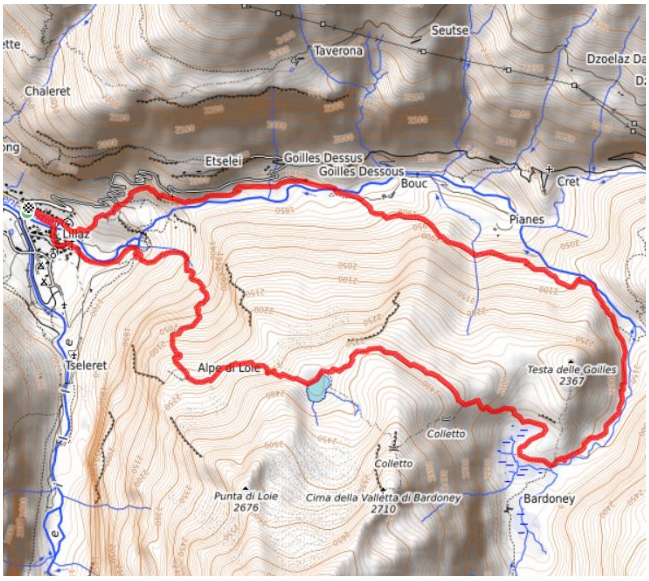

25. Pizzo d'Erna

Lombardy (Lecco)

10.2 km

857 m

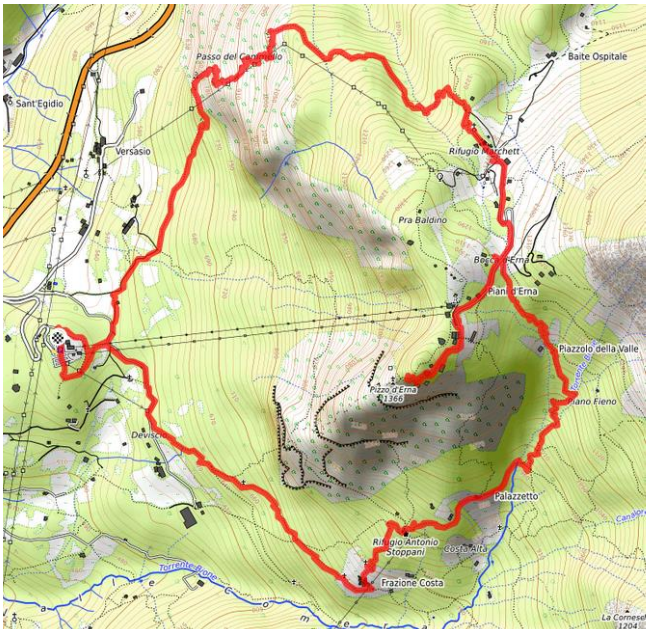

Table S1. Wikiloc hyperlinks to sources used for the study divided by trail.

1. Tre santuari di Salò	https://it.wikiloc.com/percorsi-escursionismo/tre-santuari-salo-32531618
	https://it.wikiloc.com/percorsi-escursionismo/i-tre-santuari-di-salo-renzano-24340788
	https://it.wikiloc.com/percorsi-escursionismo/da-salo-madonna-del-rio-croce-di-salo-e-san-
	https://it.wikiloc.com/percorsi-escursionismo/giro-dei-tre-santuari-da-salo-e-croce-di-san-
	https://it.wikiloc.com/percorsi-escursionismo/sentiero-dei-3-santuari-salo-easygarda-
	https://it.wikiloc.com/percorsi-escursionismo/renzano-3-santuari-di-salo-87283943
	https://it.wikiloc.com/percorsi-escursionismo/tre-santuari-attorno-a-salo-128813268
	https://it.wikiloc.com/percorsi-escursionismo/madonna-del-rio-e-s-bartolomeo-da-salo-
	https://it.wikiloc.com/percorsi-escursionismo/salo-3-santuari-81567748
	https://it.wikiloc.com/percorsi-escursionismo/renzano-anello-3-santuari-96929248
	https://it.wikiloc.com/percorsi-escursionismo/i-tre-santuari-di-salo-97759956
	https://it.wikiloc.com/percorsi-escursionismo/renzano-e-i-tre-santuari-69846870
	https://it.wikiloc.com/percorsi-escursionismo/salo-anello-dei-tre-santuari-129458046

	https://it.wikiloc.com/percorsi-escursionismo/giro-dei-tre-monasteri-85394090
	https://it.wikiloc.com/percorsi-escursionismo/giro-santuari-124584161
	https://it.wikiloc.com/percorsi-escursionismo/salo-e-dintorni-125153262
	https://it.wikiloc.com/percorsi-escursionismo/3-santuari-sopra-salo-52604724
	https://it.wikiloc.com/percorsi-escursionismo/madonna-del-rio-e-s-bartolomeo-da-renzano-
	https://it.wikiloc.com/percorsi-escursionismo/tracciato-misto-ai-3-santuari-di-salo-25166357
	https://it.wikiloc.com/percorsi-escursionismo/giro-dei-tre-santuari-di-salo-96384712
2. Sassolungo dal Passo Sella	https://it.wikiloc.com/percorsi-escursionismo/giro-del-sassolungo-108459400
	https://it.wikiloc.com/percorsi-escursionismo/sassolungo-lankofel-112132850
	https://it.wikiloc.com/percorsi-escursionismo/anello-del-sassolungo-dolomiti-val-di-fassa-
	https://it.wikiloc.com/percorsi-escursionismo/sasssolungo-3-rifugi-54747240
	https://it.wikiloc.com/percorsi-escursionismo/sassolungo-117607431
	https://it.wikiloc.com/percorsi-escursionismo/passo-sella-rifugio-toni-demetz-rifugio-
	https://it.wikiloc.com/percorsi-escursionismo/salita-al-tony-demetz-vicenza-comici-
	https://it.wikiloc.com/percorsi-escursionismo/vuelta-al-sassolungo-109034899
	https://it.wikiloc.com/percorsi-escursionismo/sassolungo-42353063
	https://it.wikiloc.com/percorsi-escursionismo/anello-sassolungo-82562125
	https://it.wikiloc.com/percorsi-escursionismo/anello-sassolungo-rifugi-demetz-vicenza-
	https://it.wikiloc.com/percorsi-escursionismo/forcella-giro-sassolungo-106898546
	https://it.wikiloc.com/percorsi-escursionismo/sassolungo-53354068
	https://it.wikiloc.com/percorsi-escursionismo/giro-anello-sassolungo-rifugio-toni-demetz-
	https://it.wikiloc.com/percorsi-escursionismo/circular-sasso-lungo-110626199
	https://it.wikiloc.com/percorsi-escursionismo/sassolungo-55478130
	https://it.wikiloc.com/percorsi-escursionismo/fossel-117280947
	https://it.wikiloc.com/percorsi-escursionismo/fossel-145656224
	https://it.wikiloc.com/percorsi-escursionismo/fossel-53491383
	https://it.wikiloc.com/percorsi-escursionismo/fossel-109938132
3. Viel del Pan	https://it.wikiloc.com/percorsi-escursionismo/passo-pordoi-viel-del-pan-porta-vescovo-
	https://it.wikiloc.com/percorsi-escursionismo/passo-pordoi-viel-del-pan-27642321
	https://it.wikiloc.com/percorsi-escursionismo/viel-del-pan-passo-pordoi-fredarola-sass-
	https://it.wikiloc.com/percorsi-escursionismo/passo-pordoi-refugio-viel-del-pan-refugio-
	https://it.wikiloc.com/percorsi-escursionismo/anello-viel-del-pan-passo-pordoi-104632488
	https://it.wikiloc.com/percorsi-escursionismo/passo-pordoi-601-viel-de-pan-680-pass-
	https://it.wikiloc.com/percorsi-escursionismo/passo-pordoi-porta-vescovo-viel-dal-pan-
	https://it.wikiloc.com/percorsi-escursionismo/anello-passo-pordoi-viel-del-pan-55330875
	https://it.wikiloc.com/percorsi-escursionismo/viel-del-pan-da-passo-pordoi-a-porta-vescovo-
	https://it.wikiloc.com/percorsi-escursionismo/passo-pordoi-viel-del-pan-porta-vescovo-
	https://it.wikiloc.com/percorsi-escursionismo/2022-07-02-dolomitas-ruta-viel-del-pan-passo-
	https://it.wikiloc.com/percorsi-escursionismo/via-del-pan-111237507
	https://it.wikiloc.com/percorsi-escursionismo/passo-pordoi-110249419
	https://it.wikiloc.com/percorsi-escursionismo/viel-del-pan-84479285
	https://it.wikiloc.com/percorsi-escursionismo/passo-pordoi-84408692
	https://it.wikiloc.com/percorsi-escursionismo/passo-pordoi-83166450
	https://it.wikiloc.com/percorsi-escursionismo/passo-pordoi-144323856
	https://it.wikiloc.com/percorsi-escursionismo/passo-pordoi-141670623

	https://it.wikiloc.com/percorsi-escursionismo/paso-pordoi-circular-82532308
	https://it.wikiloc.com/percorsi-escursionismo/marmolada-40270988
4. Piz Boè	https://it.wikiloc.com/percorsi-escursionismo/passo-pordoi-piz-boe-80789220
	https://it.wikiloc.com/percorsi-escursionismo/passo-pordoi-pitz-boe-rifugio-pitz-boe-
	https://it.wikiloc.com/percorsi-escursionismo/passo-pordoi-rif-forcella-pordoi-piz-boe-rif-
	https://it.wikiloc.com/percorsi-escursionismo/passo-pordoi-piz-boe-110826214
	https://it.wikiloc.com/percorsi-escursionismo/dolomites-2-pass-pordoi-piz-boe-110327761
	https://it.wikiloc.com/percorsi-escursionismo/passo-pordoi-54189509
	https://it.wikiloc.com/percorsi-escursionismo/passo-pordoi-piz-boe-81965530
	https://it.wikiloc.com/percorsi-escursionismo/passo-pordoi-piz-boe-78068776
	https://it.wikiloc.com/percorsi-escursionismo/passo-pordoi-piz-boe-107372518
	https://it.wikiloc.com/percorsi-escursionismo/passo-pordoi-piz-boe-110594988
	https://it.wikiloc.com/percorsi-escursionismo/passo-pordoi-piz-boe-108726629
	https://it.wikiloc.com/percorsi-escursionismo/piz-boe-rifugio-boe-da-passo-pordoi-81253596
	https://it.wikiloc.com/percorsi-escursionismo/passo-pordoi-piz-boe-andata-e-ritorno-
	https://it.wikiloc.com/percorsi-escursionismo/piz-boe-27493821
	https://it.wikiloc.com/percorsi-escursionismo/piz-boe-80867645
	https://it.wikiloc.com/percorsi-escursionismo/passo-pordoi-55120706
	https://it.wikiloc.com/percorsi-escursionismo/passo-pordoi-109731593
	https://it.wikiloc.com/percorsi-escursionismo/passo-pordoi-78957426
	https://it.wikiloc.com/percorsi-escursionismo/paso-pordoi-piz-boe-80859866
	https://it.wikiloc.com/percorsi-escursionismo/passo-pordoi-106518127
5. Settsass	https://it.wikiloc.com/percorsi-escursionismo/rifugio-valparola-giro-del-settsas-sentiero-24-
	https://it.wikiloc.com/percorsi-escursionismo/settsass-80784058
	https://it.wikiloc.com/percorsi-escursionismo/setsass-110543912
	https://it.wikiloc.com/percorsi-escursionismo/veneto-anello-del-settsass-dal-passo-valparola-
	https://it.wikiloc.com/percorsi-escursionismo/valparola-144910580
	https://it.wikiloc.com/percorsi-escursionismo/pian-falzarego-144876148
	https://it.wikiloc.com/percorsi-escursionismo/anello-settsass-53312270
	https://it.wikiloc.com/percorsi-escursionismo/settsass-giro-85876622
	https://it.wikiloc.com/percorsi-escursionismo/passa-valparola-rondje-27607281
	https://it.wikiloc.com/percorsi-escursionismo/pian-falzarego-26428199
	https://it.wikiloc.com/percorsi-escursionismo/giro-sett-sass-146133519
	https://it.wikiloc.com/percorsi-escursionismo/giro-del-settsass-dal-passo-valparola-26922075
	https://it.wikiloc.com/percorsi-escursionismo/pian-falzarego-55554892
	https://it.wikiloc.com/percorsi-escursionismo/valparola-110650055
	https://it.wikiloc.com/percorsi-escursionismo/giro-settsass-54411776
	https://it.wikiloc.com/percorsi-escursionismo/pian-falzarego-sett-sass-143289366
	https://it.wikiloc.com/percorsi-escursionismo/giro-settsass-53615984
	https://it.wikiloc.com/percorsi-escursionismo/passo-valparola-setsas-28649316
	https://it.wikiloc.com/percorsi-escursionismo/pian-falzarego-25981044
	https://it.wikiloc.com/percorsi-escursionismo/giro-intorno-al-setsas-110052445
6. Tre	https://it.wikiloc.com/percorsi-escursionismo/anello-delle-tre-cime-di-lavaredo-78859869
	https://it.wikiloc.com/percorsi-escursionismo/giro-delle-tre-cime-di-lavaredo-40592676
	https://it.wikiloc.com/percorsi-escursionismo/tre-cime-di-lavaredo-anello-orario-109581621

	https://it.wikiloc.com/percorsi-escursionismo/tre-cime-di-lavaredo-53389259
	https://it.wikiloc.com/percorsi-escursionismo/le-tre-cime-di-lavaredo-54914409
	https://it.wikiloc.com/percorsi-escursionismo/anello-delle-tre-cime-di-lavaredo-dal-rifugio-
	https://it.wikiloc.com/percorsi-escursionismo/giro-delle-tre-cime-di-lavaredo-106806856
	https://it.wikiloc.com/percorsi-escursionismo/misurina-tre-cime-di-lavaredo-39022947
	https://it.wikiloc.com/percorsi-escursionismo/tre-cime-di-lavaredo-109046014
	https://it.wikiloc.com/percorsi-escursionismo/anello-tre-cime-di-lavaredo-109412764
	https://it.wikiloc.com/percorsi-escursionismo/tre-cime-di-lavaredo-106897159
	https://it.wikiloc.com/percorsi-escursionismo/giro-delle-tre-cime-di-lavaredo-81446250
	https://it.wikiloc.com/percorsi-escursionismo/anello-tre-cime-di-lavaredo-113336273
	https://it.wikiloc.com/percorsi-escursionismo/tre-cime-di-lavaredo-passando-dal-rifugio-
	https://it.wikiloc.com/percorsi-escursionismo/circular-tre-cime-di-lavaredo-dolomitas-
	https://it.wikiloc.com/percorsi-escursionismo/tre-cime-di-lavaredo-52964820
	https://it.wikiloc.com/percorsi-escursionismo/anello-delle-tre-cime-di-lavaredo-41239529
	https://it.wikiloc.com/percorsi-escursionismo/trilho-tre-cime-di-lavaredo-105229763
	https://it.wikiloc.com/percorsi-escursionismo/tre-cime-di-lavaredo-por-rifugio-locatelli-
	https://it.wikiloc.com/percorsi-escursionismo/tre-cime-di-lavaredo-55549972
7. Lago del Sorapiss	https://it.wikiloc.com/percorsi-escursionismo/lago-sorapis-104261834
	https://it.wikiloc.com/percorsi-escursionismo/passo-tre-croci-lago-di-sorapis-110328221
	https://it.wikiloc.com/percorsi-escursionismo/passo-tre-croci-forcella-marcuoira-lago-di-
	https://it.wikiloc.com/percorsi-escursionismo/dolomitas-nuul-passo-tre-crosi-forcella-
	https://it.wikiloc.com/percorsi-escursionismo/ruta-circular-al-llac-sorapiss-82190926
	https://it.wikiloc.com/percorsi-escursionismo/passo-tre-croci-forcella-marcuoira-lago-
	https://it.wikiloc.com/percorsi-escursionismo/passo-tre-crocci-lago-di-sorapis-refugio-
	https://it.wikiloc.com/percorsi-escursionismo/passo-tre-croci-forcella-marcuoira-i-lago-
	https://it.wikiloc.com/percorsi-corsa-in-montagna/lago-sorapis-desde-trecroci-x-collado-
	https://it.wikiloc.com/percorsi-escursionismo/passo-tre-croci-cuello-lago-sorapis-passo-tre-
	https://it.wikiloc.com/percorsi-escursionismo/lago-di-sorapis-145085244
	https://it.wikiloc.com/percorsi-escursionismo/lago-di-sorapis-110945670
	https://it.wikiloc.com/percorsi-escursionismo/anello-lago-sorapis-77222025
	https://it.wikiloc.com/percorsi-escursionismo/giro-dal-passo-tre-croci-al-lago-sorapis-per-la-
	https://it.wikiloc.com/percorsi-escursionismo/lago-sorapis-belluno-da-passo-tre-croci-
	https://it.wikiloc.com/percorsi-escursionismo/giro-del-sorapis-109128753
	https://it.wikiloc.com/percorsi-escursionismo/passo-tre-croci-forcella-marcuoira-lago-
	https://it.wikiloc.com/percorsi-escursionismo/lago-di-sorapiss-40239870
	https://it.wikiloc.com/percorsi-escursionismo/passo-tre-croci-forcella-marcuoira-rif-vandelli-
	https://it.wikiloc.com/percorsi-escursionismo/al-lago-sorapiss-per-la-forcella-marcoira-
8. Passo Giau – 5 Torri	https://www.wikiloc.com/hiking-trails/5-torri-82852687
	https://www.wikiloc.com/hiking-trails/passo-giau-rifugio-averau-rifugio-nuvolau-rifugio-
	https://www.wikiloc.com/hiking-trails/anello-passo-giau-rifugio-5-torri-scoiattoli-averau-
	https://www.wikiloc.com/hiking-trails/giau-pass-112974919
	https://www.wikiloc.com/hiking-trails/rif-averau-nuvolau-scoiattoli-cinque-torri-114360911
	https://www.wikiloc.com/hiking-trails/passo-giau-cinque-torri-scoiattoli-nuvolau-averau-
	https://www.wikiloc.com/hiking-trails/passo-giau-rifugio-cinque-torri-rifugio-scoiattoli-
	https://www.wikiloc.com/hiking-trails/passo-giau-giro-delle-5-torri-106743078

	https://www.wikiloc.com/hiking-trails/paso-de-giau-cinque-torri-refugio-nuvolau-84341319
	https://www.wikiloc.com/hiking-trails/pas-del-giau-refugio-de-adelau-refugio-de-nuvolao-
	https://www.wikiloc.com/hiking-trails/posalz-108147744
	https://www.wikiloc.com/hiking-trails/passo-giau-rifugio-averau-rifugio-nuvolau-rifugio-
	https://www.wikiloc.com/hiking-trails/passo-giau-rifugio-averau-rifugio-nuvolau-5-torri-
	https://www.wikiloc.com/hiking-trails/anello-da-passo-giau-a-rif-averau-rif-nuvolau-rif-
	https://www.wikiloc.com/hiking-trails/anello-passo-giau-rifugio-averau-rifugio-nuvolau-
	https://www.wikiloc.com/hiking-trails/anello-delle-5-torri-da-passo-giau-110049363
	https://www.wikiloc.com/hiking-trails/5-rifugi-ad-anello-83709392
	https://www.wikiloc.com/hiking-trails/anello-passo-giau-rif-scoiattoli-nuvolau-averau-
	https://it.wikiloc.com/percorsi-escursionismo/anello-passo-giau-averau-nuvolau-scoiattoli-5-
	https://it.wikiloc.com/percorsi-escursionismo/passo-giau-5-torri-80090061
9. Rifugio Semenza	https://it.wikiloc.com/percorsi-escursionismo/santanna-84858958
	https://it.wikiloc.com/percorsi-escursionismo/rifugio-semenza-110625145
	https://it.wikiloc.com/percorsi-escursionismo/santanna-150041406
	https://it.wikiloc.com/percorsi-escursionismo/rifugio-semenza-38966479
	https://it.wikiloc.com/percorsi-escursionismo/santanna-54046020
	https://it.wikiloc.com/percorsi-escursionismo/semenza-110848682
	https://it.wikiloc.com/percorsi-escursionismo/rifugio-semenza-117365232
	https://it.wikiloc.com/percorsi-escursionismo/anello-tambre-malga-pian-grant-rifugio-
	https://it.wikiloc.com/percorsi-escursionismo/malga-plan-grant-rifugio-semenza-27669608
	https://it.wikiloc.com/percorsi-escursionismo/santanna-150021027
	https://it.wikiloc.com/percorsi-escursionismo/santanna-rifugio-semenza-140507175
	https://it.wikiloc.com/percorsi-escursionismo/rifugio-semenza-83659717
	https://it.wikiloc.com/percorsi-escursionismo/rifugio-semenza-sent-293-296-86681130
	https://it.wikiloc.com/percorsi-escursionismo/santanna-rifugio-semenza-87125645
	https://it.wikiloc.com/percorsi-escursionismo/malga-pian-grant-bivacco-semenza-107230215
	https://it.wikiloc.com/percorsi-escursionismo/rifugio-semenza-da-santanna-151552622
	https://it.wikiloc.com/percorsi-escursionismo/rifugio-semenza-83085888
	https://it.wikiloc.com/percorsi-escursionismo/rifugio-semenza-da-santanna-152084598
	https://it.wikiloc.com/percorsi-escursionismo/rifugio-semenza-107811775
	https://it.wikiloc.com/percorsi-escursionismo/pian-lastre-rifugio-semenza-145899959
10. San Boldo – 3 bivacchi	https://it.wikiloc.com/percorsi-escursionismo/san-boldo-bivacco-del-loff-bivacco-vallon-
	https://it.wikiloc.com/percorsi-escursionismo/passo-san-boldo-bivacco-loff-san-boldo-
	https://it.wikiloc.com/percorsi-escursionismo/passo-san-boldo-bivacco-dei-loff-cima-vallon-
	https://it.wikiloc.com/percorsi-escursionismo/san-boldo-bivacco-del-loff-san-boldo-
	https://it.wikiloc.com/percorsi-escursionismo/passo-san-boldo-sent-n-2-cima-vallonscuro-
	https://it.wikiloc.com/percorsi-escursionismo/san-boldo-cima-vallon-scuro-bivacco-loff-
	https://it.wikiloc.com/percorsi-escursionismo/da-passo-san-boldo-cima-vallon-scuro-e-
	https://it.wikiloc.com/percorsi-escursionismo/19-02-23-passo-san-boldo-bivacco-dei-loff-
	https://it.wikiloc.com/percorsi-escursionismo/san-boldo-bivacco-dei-loff-crodon-del-gevero-
	https://it.wikiloc.com/percorsi-escursionismo/san-boldo-bivacco-loff-vallon-scuro-crodon-
	https://it.wikiloc.com/percorsi-escursionismo/san-boldo-bivacco-dei-loff-78560536
	https://it.wikiloc.com/percorsi-escursionismo/bivacco-costacurta-vallon-scuro-e-loff-
	https://it.wikiloc.com/percorsi-escursionismo/san-boldo-bivacco-dei-loff-128756954

	https://it.wikiloc.com/percorsi-escursionismo/bivacco-dai-loff-61491933
	https://it.wikiloc.com/percorsi-escursionismo/san-boldo-bivacco-loff-ad-anello-72492366
	https://it.wikiloc.com/percorsi-escursionismo/san-boldo-bivacco-loff-piu-cima-130030110
	https://it.wikiloc.com/percorsi-escursionismo/san-boldo-bivacco-loft-ballon-scuro-97947249
	https://it.wikiloc.com/percorsi-escursionismo/bivacco-dei-loff-da-passo-sanboldo-
	https://it.wikiloc.com/percorsi-escursionismo/p-sso-s-boldo-bivacco-loff-cima-vallon-scuro-
	https://it.wikiloc.com/percorsi-escursionismo/san-boldo-bivacco-ai-lof-120872445
11. Malghe di Caltrano	https://it.wikiloc.com/percorsi-escursionismo/giro-delle-malghe-78081591
	https://it.wikiloc.com/percorsi-escursionismo/giro-delle-malghe-di-caltrano-113921583
	https://it.wikiloc.com/percorsi-escursionismo/malghe-56368227
	https://it.wikiloc.com/percorsi-escursionismo/giro-delle-malghe-caltrano-110166786
	https://it.wikiloc.com/percorsi-escursionismo/caltrano-giro-delle-malghe-82440293
	https://it.wikiloc.com/percorsi-escursionismo/giro-delle-malghe-altopiano-di-asiago-
	https://it.wikiloc.com/percorsi-escursionismo/giro-malghe-caltrano-115268803
	https://it.wikiloc.com/percorsi-escursionismo/giro-malghe-caltrano-110631585
	https://it.wikiloc.com/percorsi-escursionismo/giro-delle-malghe-di-calteano-110322141
	https://it.wikiloc.com/percorsi-escursionismo/giro-malghe-caltrano-81658221
	https://it.wikiloc.com/percorsi-escursionismo/giro-delle-malghe-86501018
	https://it.wikiloc.com/percorsi-escursionismo/giro-delle-malghe-cesuna-115173199
	https://it.wikiloc.com/percorsi-escursionismo/giro-delle-malghe-di-caltrano-106973241
	https://it.wikiloc.com/percorsi-escursionismo/giro-malghe-117773928
	https://it.wikiloc.com/percorsi-escursionismo/giro-malghe-bar-alpino-109322857
	https://it.wikiloc.com/percorsi-escursionismo/giro-malghe-19422596
	https://it.wikiloc.com/percorsi-escursionismo/giro-malghe-80650613
	https://it.wikiloc.com/percorsi-escursionismo/giro-malghe-caltrano-110827183
	https://it.wikiloc.com/percorsi-escursionismo/giro-delle-malghe-caltrano-82946116
	https://it.wikiloc.com/percorsi-escursionismo/giro-malghe-di-caltrano-56173472
12. Cima Zevola	https://it.wikiloc.com/percorsi-escursionismo/monte-zevola-25799384
	https://it.wikiloc.com/percorsi-escursionismo/gazza-passo-delle-tre-croci-monte-zevola-
	https://it.wikiloc.com/percorsi-escursionismo/rifugio-battisti-passi-ristele-e-tre-croci-
	https://it.wikiloc.com/percorsi-escursionismo/passo-ristele-catena-delle-tre-croci-gruppo-
	https://it.wikiloc.com/percorsi-escursionismo/monte-zevola-78082869
	https://it.wikiloc.com/percorsi-escursionismo/gazza-passo-lora-passo-ristele-86504169
	https://it.wikiloc.com/percorsi-escursionismo/monte-zevola-38951535
	https://it.wikiloc.com/percorsi-escursionismo/passo-tre-croci-e-passo-ristele-40976092
	https://it.wikiloc.com/percorsi-escursionismo/anello-zevola-e-gramolon-76940718
	https://it.wikiloc.com/percorsi-escursionismo/zevola-59584134
	https://it.wikiloc.com/percorsi-escursionismo/zevola-83790498
	https://it.wikiloc.com/percorsi-escursionismo/monte-zevola-56670473
	https://it.wikiloc.com/percorsi-escursionismo/monte-zevola-passo-ristele-53610841
	https://it.wikiloc.com/percorsi-escursionismo/turcati-8685436
	https://it.wikiloc.com/percorsi-escursionismo/giro-del-monte-zevola-83665782
	https://it.wikiloc.com/percorsi-escursionismo/gazza-ristele-lora-gazza-79116757
	https://it.wikiloc.com/percorsi-escursionismo/rifugio-cesare-battisti-79146365
	https://it.wikiloc.com/percorsi-escursionismo/recoaro-terme-rifugio-battisti-passo-tre-croci-

	https://it.wikiloc.com/percorsi-escursionismo/zevola-gramolon-59981344
	https://it.wikiloc.com/percorsi-escursionismo/monte-zevola-25799384
13. Sentiero della Cengia	https://it.wikiloc.com/percorsi-escursionismo/barbarano-vicentino-109743528
	https://it.wikiloc.com/percorsi-escursionismo/sentiero-71-della-cengia-33679601
	https://it.wikiloc.com/percorsi-escursionismo/barbarano-vicentino-60507692
	https://it.wikiloc.com/percorsi-escursionismo/barbarano-vicentino-zovencedo-119848245
	https://it.wikiloc.com/percorsi-escursionismo/sentiero-n71-della-cengia-aggiornato-
	https://it.wikiloc.com/percorsi-escursionismo/sentiero-della-cengia-n-71-136739419
	https://it.wikiloc.com/percorsi-escursionismo/n-71-da-barbarano-vicentino-60506950
	https://it.wikiloc.com/percorsi-escursionismo/barbarano-vicentino-cengia-69867839
	https://it.wikiloc.com/percorsi-escursionismo/la-via-della-cengia-119008709
	https://it.wikiloc.com/percorsi-escursionismo/barbarano-vicentino-71-87244214
	https://it.wikiloc.com/percorsi-escursionismo/barbarano-vicentino-94917947
	https://it.wikiloc.com/percorsi-escursionismo/barbarano-vicentino-64211507
	https://it.wikiloc.com/percorsi-escursionismo/barbarano-vicentino-85821875
	https://it.wikiloc.com/percorsi-escursionismo/barbarano-vicentino-133247064
	https://it.wikiloc.com/percorsi-escursionismo/barbarano-vicentino-67365810
	https://it.wikiloc.com/percorsi-escursionismo/barbarano-vicentino-75777910
	https://it.wikiloc.com/percorsi-escursionismo/barbarano-vicentino-118307242
	https://it.wikiloc.com/percorsi-escursionismo/barbarano-vicentino-62610367
	https://it.wikiloc.com/percorsi-escursionismo/barbarano-vicentino-124548055
	https://it.wikiloc.com/percorsi-escursionismo/sentiero-71-della-cengia-22590979
14. Monte Venda	https://it.wikiloc.com/percorsi-escursionismo/sentiero-lorenzoni-25493211
	https://it.wikiloc.com/percorsi-escursionismo/monte-venda-e-santuario-96180288
	https://it.wikiloc.com/percorsi-escursionismo/fin-sugli-olivetani-140549474
	https://it.wikiloc.com/percorsi-escursionismo/monte-venda-olivetani-66845680
	https://it.wikiloc.com/percorsi-escursionismo/faedo-81063503
	https://it.wikiloc.com/percorsi-escursionismo/monte-rua-131195128
	https://it.wikiloc.com/percorsi-escursionismo/monte-venda-corno-del-venda-129617564
	https://it.wikiloc.com/percorsi-escursionismo/monte-rua-monte-venda-monasterio-oliveti-
	https://it.wikiloc.com/percorsi-escursionismo/monte-rua-53404580
	https://it.wikiloc.com/percorsi-escursionismo/faedo-135892758
	https://it.wikiloc.com/percorsi-escursionismo/alta-via-sui-colli-euganei-76871936
	https://it.wikiloc.com/percorsi-escursionismo/monte-venda-118295643
	https://it.wikiloc.com/percorsi-escursionismo/monte-venda-casa-marina-76871869
	https://it.wikiloc.com/percorsi-escursionismo/per-gli-olivetani-97651860
	https://it.wikiloc.com/percorsi-escursionismo/monte-venda-34897574
	https://it.wikiloc.com/percorsi-escursionismo/sentiero-del-monte-venda-g-g-lorenzoni-
	https://it.wikiloc.com/percorsi-escursionismo/giro-del-monte-venda-96956277
	https://it.wikiloc.com/percorsi-escursionismo/faedo-89272699
	https://it.wikiloc.com/percorsi-escursionismo/giro-del-monte-venda-36055399
	https://it.wikiloc.com/percorsi-escursionismo/monte-rua-53415295
15. Malghe di [illegible]	https://it.wikiloc.com/percorsi-escursionismo/giro-delle-malghe-versione-1-piancavallo-
	https://it.wikiloc.com/percorsi-escursionismo/piancavallo-passeggiata-delle-malghe-
	https://it.wikiloc.com/percorsi-escursionismo/giro-delle-malghe-piancavallo-59259414

	https://it.wikiloc.com/percorsi-escursionismo/giro-delle-malghe-piancavallo-da-via-collalto-
	https://it.wikiloc.com/percorsi-escursionismo/giro-malghe-piancavallo-111398477
	https://it.wikiloc.com/percorsi-escursionismo/piancavallo-passeggiata-delle-malghe-
	https://it.wikiloc.com/percorsi-escursionismo/passeggiata-delle-malghe-dal-rampy-park-
	https://it.wikiloc.com/percorsi-escursionismo/giro-delle-malghe-106878955
	https://it.wikiloc.com/percorsi-escursionismo/giro-delle-malghe-piancavallo-82923900
	https://it.wikiloc.com/percorsi-escursionismo/piancavallo-anelli-delle-malghe-98460351
	https://it.wikiloc.com/percorsi-escursionismo/piancavallo-giro-delle-malghe-122887900
	https://it.wikiloc.com/percorsi-escursionismo/piancavallo-giro-delle-malghe-123516733
	https://it.wikiloc.com/percorsi-escursionismo/passeggiata-delle-malghe-piancavallo-
	https://it.wikiloc.com/percorsi-escursionismo/piancavallo-111955665
	https://it.wikiloc.com/percorsi-escursionismo/giro-delle-malghe-piancavallo-medio-
	https://it.wikiloc.com/percorsi-escursionismo/giro-delle-malghe-piancavallo-119071338
	https://it.wikiloc.com/percorsi-escursionismo/sentiero-delle-malghe-40118528
	https://it.wikiloc.com/percorsi-escursionismo/passeggiata-delle-malghe-37610665
	https://it.wikiloc.com/percorsi-escursionismo/piancavallo-43099336
	https://it.wikiloc.com/percorsi-escursionismo/piancavallo-148110076
16. Monte Autore	https://it.wikiloc.com/percorsi-escursionismo/anello-monte-autore-55054144
	https://it.wikiloc.com/percorsi-escursionismo/anello-campo-dellosso-monte-autore-
	https://it.wikiloc.com/percorsi-escursionismo/anello-del-monte-autore-da-campo-dellosso-
	https://it.wikiloc.com/percorsi-escursionismo/anello-monte-autore-74750527
	https://it.wikiloc.com/percorsi-escursionismo/anello-del-monte-autore-da-campo-dellosso-e-
	https://it.wikiloc.com/percorsi-escursionismo/anello-monte-autore-94088419
	https://it.wikiloc.com/percorsi-escursionismo/monte-autore-ad-anello-da-campo-dellosso-
	https://it.wikiloc.com/percorsi-escursionismo/anello-del-monte-autore-20919446
	https://it.wikiloc.com/percorsi-escursionismo/anello-campo-dellosso-monte-autore-
	https://it.wikiloc.com/percorsi-escursionismo/monte-autore-08-07-2017-18557934
	https://it.wikiloc.com/percorsi-escursionismo/monte-autore-da-campo-dellosso-117757915
	https://it.wikiloc.com/percorsi-escursionismo/anello-del-monte-autore-59091065
	https://it.wikiloc.com/percorsi-escursionismo/monte-autore-da-campo-dellosso-100454945
	https://it.wikiloc.com/percorsi-escursionismo/anello-monte-autore-103550190
	https://it.wikiloc.com/percorsi-escursionismo/monte-autore-parco-dei-monti-simbruini-
	https://it.wikiloc.com/percorsi-escursionismo/monte-livata-amp-monte-autore-simbruini-
	https://it.wikiloc.com/percorsi-escursionismo/anello-campo-dellosso-monte-autore-
	https://it.wikiloc.com/percorsi-escursionismo/anello-campo-dellosso-monte-autore-monna-
	https://it.wikiloc.com/percorsi-escursionismo/monte-autore-anello-74743522
	https://it.wikiloc.com/percorsi-escursionismo/anello-campo-dellosso-monte-autore-
17. Monte Venere	https://it.wikiloc.com/percorsi-escursionismo/monte-venere-66821603
	https://it.wikiloc.com/percorsi-escursionismo/monte-venere-pozzo-del-diavolo-65461787
	https://it.wikiloc.com/percorsi-escursionismo/percorso-ad-anello-monte-venere-18489860
	https://it.wikiloc.com/percorsi-escursionismo/localita-canale-monte-venere-pozzo-del-
	https://it.wikiloc.com/percorsi-escursionismo/bellezza-e-avventura-nella-faggeta-del-monte-
	https://it.wikiloc.com/percorsi-escursionismo/fb-monte-venere-122804492
	https://it.wikiloc.com/percorsi-escursionismo/2017-10-14-monte-venere-lago-di-vico-
	https://it.wikiloc.com/percorsi-escursionismo/monte-venere-e-pozzo-del-diavolo-anello-

	https://it.wikiloc.com/percorsi-escursionismo/monte-venere-41807548
	https://it.wikiloc.com/percorsi-escursionismo/monte-venere-pozzo-del-diavolo-101798406
	https://it.wikiloc.com/percorsi-escursionismo/monte-venere-e-pizzo-del-diavolo-40703888
	https://it.wikiloc.com/percorsi-escursionismo/monte-venere-e-pozzo-del-diavolo-59702131
	https://it.wikiloc.com/percorsi-escursionismo/monte-venere-pozzo-del-diavolo-74523742
	https://it.wikiloc.com/percorsi-escursionismo/monte-venere-128a-128b-104636919
	https://it.wikiloc.com/percorsi-escursionismo/monte-venere-114479713
	https://it.wikiloc.com/percorsi-escursionismo/monte-venere-84193837
	https://it.wikiloc.com/percorsi-escursionismo/sentiero-monte-venere-111823512
	https://it.wikiloc.com/percorsi-escursionismo/faggeta-monte-venere-80658528
	https://it.wikiloc.com/percorsi-escursionismo/monte-venere-gola-del-diavolo-87257979
	https://it.wikiloc.com/percorsi-escursionismo/da-fonte-canale-a-monte-venere-87246399
18. Grotte di Labante	https://it.wikiloc.com/percorsi-escursionismo/grotte-di-labante-86714692
	https://it.wikiloc.com/percorsi-escursionismo/grotte-di-labante-mulino-povolo-grotte-le-tane-
	https://it.wikiloc.com/percorsi-escursionismo/grotte-di-labante-e-sentiero-delle-tane-
	https://it.wikiloc.com/percorsi-escursionismo/grotte-di-labante-le-tane-118330537
	https://it.wikiloc.com/percorsi-escursionismo/grotte-di-labante-104639150
	https://it.wikiloc.com/percorsi-escursionismo/grotte-di-labante-93999306
	https://it.wikiloc.com/percorsi-escursionismo/cascata-di-labante-73765265
	https://it.wikiloc.com/percorsi-escursionismo/labante-cai-152a-162-51851066
	https://it.wikiloc.com/percorsi-escursionismo/grotta-di-labante-78548564
	https://it.wikiloc.com/percorsi-escursionismo/cascata-del-labante-via-delle-tane-cascata-del-
	https://it.wikiloc.com/percorsi-escursionismo/grotta-di-labante-e-tane-nel-bosco-82486025
	https://it.wikiloc.com/percorsi-escursionismo/cascate-di-labante-100463534
	https://it.wikiloc.com/percorsi-escursionismo/grotta-labante-57163292
	https://it.wikiloc.com/percorsi-escursionismo/labantesentiero-delle-tane-79651452
	https://it.wikiloc.com/percorsi-escursionismo/percorso-tane-cascate-del-labante-75115870
	https://it.wikiloc.com/percorsi-escursionismo/labante-101041057
	https://it.wikiloc.com/percorsi-escursionismo/labante-60067340
	https://it.wikiloc.com/percorsi-escursionismo/labante-128726337
	https://it.wikiloc.com/percorsi-escursionismo/labante-masare-60021430
	https://it.wikiloc.com/percorsi-escursionismo/grotte-82476419
19. Corno alle Scale	https://it.wikiloc.com/percorsi-escursionismo/corno-alle-scale-cupolino-lago-scafaiolo-
	https://it.wikiloc.com/percorsi-escursionismo/corno-alle-scale-dai-balzi-dellora-56077077
	https://it.wikiloc.com/percorsi-escursionismo/anello-di-corno-alle-scale-49478032
	https://it.wikiloc.com/percorsi-escursionismo/corno-alle-scale-anello-37174613
	https://it.wikiloc.com/percorsi-escursionismo/anello-del-corno-alle-scale-e-cascate-del-
	https://it.wikiloc.com/percorsi-escursionismo/parco-del-corno-alle-scale-l-bo-77429028
	https://it.wikiloc.com/percorsi-escursionismo/dal-rifugio-cavone-mt-spigolino-lago-
	https://it.wikiloc.com/percorsi-escursionismo/cavone-balzo-dellora-corno-alle-scale-lago-
	https://it.wikiloc.com/percorsi-escursionismo/corno-alle-scale-rifugio-cavone-punta-sofia-
	https://it.wikiloc.com/percorsi-escursionismo/rifugio-cavone-corno-alle-scale-lago-
	https://it.wikiloc.com/percorsi-escursionismo/laghetto-cabine-balsi-dellora-corno-alle-scale-
	https://it.wikiloc.com/percorsi-escursionismo/corno-alle-scale-cavone-croce-punta-sofia-
	https://it.wikiloc.com/percorsi-escursionismo/corno-alle-scale-56649839

	https://it.wikiloc.com/percorsi-escursionismo/anello-cascate-del-dardagna-lago-scaffaiolo-
	https://it.wikiloc.com/percorsi-escursionismo/lago-di-cavone-corno-alle-scale-creste-cascate-
	https://it.wikiloc.com/percorsi-escursionismo/anello-cascate-del-dardagna-lago-scaffaiolo-
	https://it.wikiloc.com/percorsi-escursionismo/corno-alle-scale-lago-scaffaiolo-monte-
	https://it.wikiloc.com/percorsi-escursionismo/corno-alle-scale-80707739
	https://it.wikiloc.com/percorsi-escursionismo/poggiolforato-croce-corno-alle-scale-89276543
	https://it.wikiloc.com/percorsi-escursionismo/corno-alle-scale-38941849
20. San Pellegrino in Alpe	https://it.wikiloc.com/percorsi-escursionismo/san-pellegrino-in-alpe-101429993
	https://it.wikiloc.com/percorsi-escursionismo/san-pellegrino-in-alpe-il-giro-del-diavolo-
	https://it.wikiloc.com/percorsi-escursionismo/san-pellegrino-in-alpe-giro-del-diavolo-
	https://it.wikiloc.com/percorsi-escursionismo/san-pellegrino-in-alpe-giro-del-diavolo-
	https://it.wikiloc.com/percorsi-escursionismo/san-pellegrino-in-alpe-giro-del-giavolo-
	https://it.wikiloc.com/percorsi-escursionismo/san-pellegrino-in-alpe-122368622
	https://it.wikiloc.com/percorsi-escursionismo/san-pellegrino-in-alpe-24306259
	https://it.wikiloc.com/percorsi-escursionismo/san-pellegrino-in-alpe-56255645
	https://it.wikiloc.com/percorsi-escursionismo/p-lagadello-alpe-di-san-pellegrino-m-spicchio-
	https://it.wikiloc.com/percorsi-escursionismo/passo-del-lagadello-giro-del-diavolo-monte-
	https://it.wikiloc.com/percorsi-escursionismo/san-pellegrino-in-alpe-rif-burigone-78655610
	https://it.wikiloc.com/percorsi-escursionismo/ciaspolata-san-pellegrino-in-alpe-125029971
	https://it.wikiloc.com/percorsi-escursionismo/sanpellegrino-in-alpe-monte-spicchio-i-sassi-
	https://it.wikiloc.com/percorsi-escursionismo/san-pellegrino-in-alpe-94498641
	https://it.wikiloc.com/percorsi-escursionismo/san-pellegrino-in-alpe-105860802
	https://it.wikiloc.com/percorsi-escursionismo/san-pellegrino-in-alpe-130085767
	https://it.wikiloc.com/percorsi-escursionismo/giro-del-diavolo-51339120
	https://it.wikiloc.com/percorsi-escursionismo/giro-del-diavolo-versione-di-enrico-107020967
	https://it.wikiloc.com/percorsi-escursionismo/san-pellegrinetto-burigone-di-cresta-e-ritorno-
	https://it.wikiloc.com/percorsi-escursionismo/giro-del-diavolo-39886704
21. Anello Marola - Campiglia	https://it.wikiloc.com/percorsi-escursionismo/campiglia-schiara-monesteroli-fossola-
	https://it.wikiloc.com/percorsi-escursionismo/campiglia-92329487
	https://it.wikiloc.com/percorsi-escursionismo/campiglia-94519341
	https://it.wikiloc.com/percorsi-escursionismo/campiglia-119795787
	https://it.wikiloc.com/percorsi-escursionismo/anello-campiglia-momesteroli-49485246
	https://it.wikiloc.com/percorsi-escursionismo/campiglia-monasteroli-stairway-to-heaven-
	https://it.wikiloc.com/percorsi-escursionismo/campiglia-monesteroli-fossola-s-antonio-
	https://it.wikiloc.com/percorsi-escursionismo/anello-di-campiglia-sentieri-delle-cinque-terre-
	https://it.wikiloc.com/percorsi-escursionismo/campiglia-monesteroli-fossola-campiglia-
	https://it.wikiloc.com/percorsi-escursionismo/campiglia-monesteroli-anello-98373053
	https://it.wikiloc.com/percorsi-escursionismo/anello-campiglia-monesteroli-98373539
	https://it.wikiloc.com/percorsi-escursionismo/campiglia-monasteroli-campiglia-101121430
	https://it.wikiloc.com/percorsi-escursionismo/campiglia-47637005
	https://it.wikiloc.com/percorsi-escursionismo/campiglia-monesteroli-43109189
	https://it.wikiloc.com/percorsi-escursionismo/campiglia-monasteroli-fossa-92968635
	https://it.wikiloc.com/percorsi-escursionismo/campiglia-67239580
	https://it.wikiloc.com/percorsi-escursionismo/campiglia-monesteroli-fossola-32877748
	https://it.wikiloc.com/percorsi-escursionismo/campiglia-s-antonio-fossola-monesteroli-

	https://it.wikiloc.com/percorsi-escursionismo/campiglia-monasteroli-fossola-campiglia-
	https://it.wikiloc.com/percorsi-escursionismo/campiglia-92455807
22. Tre cime del Bondone	https://it.wikiloc.com/percorsi-escursionismo/monte-bondone-59695186
	https://it.wikiloc.com/percorsi-escursionismo/3-cime-74832230
	https://it.wikiloc.com/percorsi-escursionismo/3-cime-del-bondone-25656565
	https://it.wikiloc.com/percorsi-escursionismo/rifugio-viote-3-cime-82142548
	https://it.wikiloc.com/percorsi-escursionismo/tre-cime-del-bondone-e-la-ferrata-giulio-
	https://it.wikiloc.com/percorsi-escursionismo/tre-cime-del-bondone-11365647
	https://it.wikiloc.com/percorsi-escursionismo/tre-cime-del-bondone-25663121
	https://it.wikiloc.com/percorsi-escursionismo/tre-cime-di-bondone-117819147
	https://it.wikiloc.com/percorsi-escursionismo/3-cime-del-bondone-25656565
	https://it.wikiloc.com/percorsi-escursionismo/tre-cime-61266775
	https://it.wikiloc.com/percorsi-escursionismo/le-tre-cime-bondone-107593785
	https://it.wikiloc.com/percorsi-escursionismo/tre-cime-bondone-cornetto-cima-verde-
	https://it.wikiloc.com/percorsi-escursionismo/le-tre-cime-del-bondone-81264337
	https://it.wikiloc.com/percorsi-escursionismo/tre-cime-bondone-20733839
	https://it.wikiloc.com/percorsi-escursionismo/anello-delle-tre-cime-del-monte-bondone-
	https://it.wikiloc.com/percorsi-escursionismo/tre-cime-del-bondone-112696581
	https://it.wikiloc.com/percorsi-escursionismo/anello-delle-3-cime-del-bondone-81060302
	https://it.wikiloc.com/percorsi-escursionismo/3-cime-del-bondone-80377453
	https://it.wikiloc.com/percorsi-escursionismo/3-cime-bondone-108441527
	https://it.wikiloc.com/percorsi-escursionismo/3-cime-bondone-104425015
23. Monte Scaletta	https://it.wikiloc.com/percorsi-escursionismo/tour-scaletta-78957616
	https://it.wikiloc.com/percorsi-escursionismo/anello-monte-scaletta-108460436
	https://it.wikiloc.com/percorsi-escursionismo/monte-scaletta-valle-maira-108882141
	https://it.wikiloc.com/percorsi-escursionismo/monte-scaletta-da-viviere-108526400
	https://it.wikiloc.com/percorsi-escursionismo/anello-monte-scaletta-78281081
	https://it.wikiloc.com/percorsi-escursionismo/anello-monte-scaletta-22-105242373
	https://it.wikiloc.com/percorsi-escursionismo/monte-scaletta-rocca-peroni-sentiero-roberto-
	https://it.wikiloc.com/percorsi-escursionismo/monte-scaletta-85405318
	https://it.wikiloc.com/percorsi-escursionismo/monte-scaletta-2840m-52674321
	https://it.wikiloc.com/percorsi-escursionismo/monte-scaletta-108732042
	https://it.wikiloc.com/percorsi-escursionismo/pratolungo-passo-scaletta-passo-peroni-
	https://it.wikiloc.com/percorsi-escursionismo/monte-scaletta-51475643
	https://it.wikiloc.com/percorsi-escursionismo/anello-sul-monte-scaletta-106746039
	https://it.wikiloc.com/percorsi-escursionismo/anello-scaletta-23-139933317
	https://it.wikiloc.com/percorsi-escursionismo/monte-scaletta-da-viviere-prato-ciorlieta-
	https://it.wikiloc.com/percorsi-escursionismo/monte-scaletta-da-viviere-139419349
	https://it.wikiloc.com/percorsi-escursionismo/punta-scaletta-sentiero-cavallero-110738390
	https://it.wikiloc.com/percorsi-escursionismo/scaletta-mio-145180436
	https://it.wikiloc.com/percorsi-escursionismo/giro-del-monte-scaletta-passo-peroni-colletta-
	https://it.wikiloc.com/percorsi-escursionismo/monte-scaletta-e-2-valli-109963272
24. Lago Loie	https://it.wikiloc.com/percorsi-escursionismo/circular-lillaz-cascada-lillaz-lago-loie-
	https://it.wikiloc.com/percorsi-escursionismo/lillaz-53266283
	https://it.wikiloc.com/percorsi-escursionismo/lillaz-lago-delle-loie-bardoney-lillaz-81881653

	https://it.wikiloc.com/percorsi-escursionismo/lillaz-lago-loie-108310609
	https://it.wikiloc.com/percorsi-escursionismo/lilla-lago-di-loie-lillaz-26199857
	https://it.wikiloc.com/percorsi-escursionismo/lillaz-lago-loie-104607276
	https://it.wikiloc.com/percorsi-escursionismo/lillaz-lago-delle-loje-lillaz-112338117
	https://it.wikiloc.com/percorsi-escursionismo/lillaz-lac-de-loie-lillaz-81559890
	https://it.wikiloc.com/percorsi-escursionismo/lillaz-27866620
	https://it.wikiloc.com/percorsi-escursionismo/lillaz-lac-del-loie-53742534
	https://it.wikiloc.com/percorsi-escursionismo/lullaz-139564694
	https://it.wikiloc.com/percorsi-escursionismo/cascate-lillaz-lago-de-loie-bardoney-40321562
	https://it.wikiloc.com/percorsi-escursionismo/lillaz-e-alpe-di-loie-140760098
	https://it.wikiloc.com/percorsi-escursionismo/lillaz-140732778
	https://it.wikiloc.com/percorsi-escursionismo/lillaz-italie-114174892
	https://it.wikiloc.com/percorsi-escursionismo/lillaz-81357510
	https://it.wikiloc.com/percorsi-escursionismo/giorno-6-82844298
	https://it.wikiloc.com/percorsi-escursionismo/lillaz-141595260
	https://it.wikiloc.com/percorsi-escursionismo/lillaz-146128920
	https://it.wikiloc.com/percorsi-escursionismo/lillaz-147592517
25. Pizzo d'Erna	https://it.wikiloc.com/percorsi-escursionismo/erna-anello-40291078
	https://it.wikiloc.com/percorsi-escursionismo/malnago-61009793
	https://it.wikiloc.com/percorsi-escursionismo/deviscio-malnago-112733427
	https://it.wikiloc.com/percorsi-escursionismo/anello-al-pizzo-derna-6932880
	https://it.wikiloc.com/percorsi-escursionismo/croce-del-pizzo-derna-piani-derna-74357177
	https://it.wikiloc.com/percorsi-escursionismo/giro-ad-anello-piazzale-funivia-pizzo-derna-
	https://it.wikiloc.com/percorsi-escursionismo/wip-pizzo-derna-1373m-partenza-piazzale-
	https://it.wikiloc.com/percorsi-escursionismo/anello-piani-derna-102657025
	https://it.wikiloc.com/percorsi-escursionismo/versasio-piani-derna-106029445
	https://it.wikiloc.com/percorsi-escursionismo/anello-del-pizzo-derna-dal-passo-del-
	https://it.wikiloc.com/percorsi-escursionismo/piani-derna-rifugio-stoppani-rifugio-marchett-
	https://it.wikiloc.com/percorsi-escursionismo/piani-derna-85873804
	https://it.wikiloc.com/percorsi-escursionismo/lecco-piani-derna-38190966
	https://it.wikiloc.com/percorsi-escursionismo/versasio-pizzo-derna-94947740
	https://it.wikiloc.com/percorsi-escursionismo/fun-versasio-rif-stoppani-rif-marchett-fun-
	https://it.wikiloc.com/percorsi-escursionismo/malnago-109937904
	https://it.wikiloc.com/percorsi-escursionismo/malnago-27495460
	https://it.wikiloc.com/percorsi-escursionismo/anello-piani-derna-49858785
	https://it.wikiloc.com/percorsi-escursionismo/deviscio-piani-derna-55043375
	https://it.wikiloc.com/percorsi-escursionismo/versasio-71346167

ST2. Comparison of real uploaded hiking times (Wikiloc) and selected estimation methods.

Trail	Wikiloc hh:mm)	Komoot (hh:mm)	ΔWK (min)	p	Outdoor active (hh:mm)	ΔWO (min)	p	Mountain signs (hh:mm)	ΔWS (min)	p	MOVE (hh:mm)	ΔWM (min)	p
Trail #1	2:39 ± 39	3:14 ± 15	-35.00 ± 37.19	0.001	4:03 ± 15	-83.60 ± 42.88	<0.001	2:10	29.15 ± 39.45	0.003	2:26 ± 16	13.30 ± 41.32	0.456
Trail #2	3:29 ± 37	4:43 ± 10	-74.65 ± 37.84	<0.001	4:57 ± 35	-88.25 ± 36.62	<0.001	4:30	-61.25 ± 36.89	<0.001	3:38 ± 37	-9.65 ± 49.03	0.433
Trail #3	3:36 ± 44	4:20 ± 14	-44.20 ± 43.86	0.001	4:42 ± 31	-65.75 ± 43.12	<0.001	4:00	-24.25 ± 44.09	0.030	3:48 ± 31	-12.35 ± 54.73	0.370
Trail #4	3:23 ± 61	4:54 ± 18	-90.75 ± 58.34	<0.001	5:04 ± 23	-100.70 ± 67.58	<0.001	5:30	-126.95 ± 60.91	<0.001	4:33 ± 58	-69.50 ± 72.90	0.001
Trail #5	3:31 ± 21	4:23 ± 4	-51.35 ± 22.41	<0.001	3:46 ± 11	-14.20 ±23.06	0.013	3:30	1.30 ± 21.45	0.478	2:49 ± 17	42.10 ± 23.41	<0.001
Trail #6	3:03 ± 24	3:28 ± 8	-25.50 ± 25.05	<0.001	3:20 ± 9	-17.25 ± 25.24	0.011	3:00	2.50 ± 24.36	0.809	2:39 ± 24	23.05 ± 30.89	0.006
Trail #7	3:58 ± 58	5:27 ± 4	-89.00 ± 58.84	<0.001	5:33 ± 22	-94.80 ± 51.96	<0.001	4:30	-32.30 ± 57.94	0.008	3:47 ± 29	10.50 ± 70.22	0.709
Trail #8	3:43 ± 42	4:03 ± 9	-19.50 ± 45.11	0.076	5:03 ± 22	-79.80 ± 49.55	<0.001	3:30	13.20 ± 42.47	0.218	4:08 ± 49	-24.55 ± 59.52	0.100
Trail #9	3:20 ± 38	4:51 ± 22	-90.35 ± 37.13	<0.001	4:35 ± 11	-74.85 ± 38.23	<0.001	4:30	-69.85 ± 38.03	<0.001	3:11 ± 31	9.35 ± 44.52	0.350
Trail #10	2:47 ± 38	3:42 ± 14	-55.30 ± 35.62	<0.001	4:50 ± 29	-123.15 ± 31.63	<0.001	3:30	-43.40 ± 37.94	<0.001	3:05 ± 32	-18.00 ± 39.91	0.046
Trail #11	3:12 ± 67	3:32 ± 14	-20.30 ± 59.89	0.032	3:48 ± 41	-35.65 ± 67.96	0.008	3:00	11.85 ± 66.51	0.513	3:36 ± 64	-23.95 ± 76.78	0.100
Trail #12	3:02 ± 37	3:50 ± 23	-48.25 ± 42.48	0.002	4:06 ± 31	-63.40 ± 37.15	<0.001	3:30	-27.90 ± 36.53	0.006	2:50 ± 22	12.35 ± 26.31	0.019

Trail #13	2:23 ± 24	3:11 ± 5	-48.75 ± 24.64	<0.001	3:46 ± 15	-83.15 ± 25.50	<0.001	3:00	-37.40 ± 23.58	<0.001	2:36 ± 29	-13.80 ± 27.33	0.033
Trail #14	1:55 ± 29	2:43 ± 8	-48.00 ± 29.17	<0.001	3:25 ± 14	-89.85 ± 27.87	<0.001	3:00	-64.85 ± 28.61	<0.001	2:11 ± 35	-15.55 ± 46.19	0.184
Trail #15	2:09 ± 18	2:26 ± 9	-17.50 ± 17.72	0.001	2:38 ± 14	-28.65 ± 16.62	<0.001	2:15	-6.15 ± 18.08	0.150	2:01 ± 16	7.80 ± 21.03	0.100
Trail #16	2:33 ± 33	2:45 ± 13	-11.75 ± 39.32	0.126	2:48 ± 9	-14.85 ± 35.68	0.021	2:10	23.40 ± 33.05	0.005	2:05 ± 11	28.05 ± 38.82	0.003
Trail #17	1:40 ± 58	1:33 ± 6	6.90 ± 57.61	0.313	1:51 ± 12	-11.55 ± 57.61	0.020	1:40	-0.30 ± 58.17	0.062	1:12 ± 9	27.70 ± 58.49	0.007
Trail #18	2:20 ± 31	2:34 ± 5	-14.15 ± 30.54	0.051	3:08 ± 14	-48.05 ± 32.13	<0.001	2:10	10.20 ± 30.79	0.145	2:02 ± 11	18.05 ± 29.63	0.007
Trail #19	4:39 ± 84	5:13 ± 21	-33.90 ± 79.98	0.033	5:55 ± 34	-76.00 ± 74.18	0.001	5:30	-51.25 ± 83.73	0.021	4:30 ± 82	8.85 ± 108.99	0.411
Trail #20	3:29 ± 36	3:53 ± 16	-23.75 ± 37.69	0.015	4:15 ± 23	-46.10 ± 36.46	<0.001	4:00	-30.85 ± 36.34	0.005	3:00 ± 23	29.00 ± 43.16	0.009
Trail #21	3:07 ± 71	3:03 ± 14	3.68 ± 66.83	0.911	4:14 ± 24	-68.21 ± 70.65	0.002	3:30	-23.74 ± 73.18	0.116	2:33 ± 18	34.70 ± 71.04	0.065
Trail #22	3:29 ± 63	4:37 ± 12	-68.25 ± 61.17	0.002	4:24 ± 14	-54.85 ± 65.50	0.006	4:00	-31.10 ± 63.16	0.012	3:08 ± 29	20.65 ± 74.36	0.279
Trail #23	4:11 ± 44	6:24 ± 27	-133.45 ± 46.63	<0.001	6:19 ± 37	-128.55 ± 57.92	<0.001	5:30	-79.30 ± 43.83	<0.001	5:13 ± 134	-61.90 ± 142.82	0.040
Trail #24	3:37 ± 41	5:20 ± 10	-103.35 ± 38.17	<0.001	5:21 ± 25	-104.15 ± 49.02	<0.001	4:30	-53.15 ± 41.14	<0.001	4:06 ± 36	-29.55 ± 56.81	0.038
Trail #25	2:53 ± 65	4:21 ± 15	-87.50 ± 61.56	<0.001	5:07 ± 32	-133.50 ± 57.11	<0.001	4:00	-67.00 ± 65.20	0.001	3:06 ± 24	-13.30 ± 59.74	0.411
**Whole sample (n=500)**	**3:07 ± 62**	**3:56 ± 67**	**-48.92 ± 57.16**	**<0.001**	**4:16 ± 66**	**-69.13 ± 58.23**	**<0.001**	**3:37 ± 63**	**-29.59 ± 59.90**	**<0.001**	**3:07 ± 69**	**-0.27 ± 65.72**	**0.278**
